# APOE ε4 allele drives female-specific Alzheimer’s disease progression via vascular dysfunction and tau spreading

**DOI:** 10.3389/fnins.2025.1683204

**Published:** 2025-12-02

**Authors:** Shuai Huang, Jiawei Wu, Ling He

**Affiliations:** 1 Department of Pharmacology, School of Pharmacy, China Pharmaceutical University, Nanjing, China; 2 Department of Gastroenterology, Affiliated Kunshan Hospital of Jiangsu University, Kunshan, China

**Keywords:** APOE ε4 allele, Alzheimer’s disease, sex, tau, endothelial cells

## Abstract

**Background:**

Apolipoprotein E (APOE) epsilon4 (ε4) is a major genetic risk factor for late-onset Alzheimer’s disease (AD), with women exhibiting heightened vulnerability to APOE ε4-associated cognitive impairment. Despite recognition of this sexual dimorphism, the underlying biological mechanisms remain incompletely understood.

**Methods:**

We performed weighted gene co-expression network analysis (WGCNA) on RNA-seq data from the Mayo Clinic cohort (*n* = 277 temporal cortex samples) to identify sex- and APOE ε4-linked co-expression modules. 315 hub genes were identified within the most relevant gene modules derived from female AD patients with APOE ε4. The expression patterns of representative hub genes were then validated in female APOE ε4 carriers. Causal genes were prioritized via Summary-data-based Mendelian Randomization (SMR), and diagnostic biomarkers were identified using machine learning. Single-cell RNA-seq elucidated cell-type-specific gene expression, and Connectivity Map (CMap) screening nominated candidate therapeutics validated in a tauopathy mouse model (AAV-hTau-injected APP/PS1 mice).

**Results:**

Four co-expression modules (*tan*, *blue2*, *grey60*, *antiquewhite4*) specifically correlated with female AD patients with APOE ε4-positive and were enriched for vascular endothelial development and extracellular matrix pathways. Ten hub genes were implicated in the pathogenesis of female-specific APOE ε4 AD. *LAMC1*, *RBMS2*, *TMOD3*, and *LRP10* were suggested as key drivers of AD progression associated with endothelial dysfunction. Single-cell analysis confirmed endothelial-specific upregulation of these genes in female APOE ε4 AD brains. Drug repositioning nominated the vasodilator vincamine, which downregulated *Lrp10*, *Lamc1* in cortex tissue, and effectively inhibited the tau protein propagation from the medial entorhinal cortex (MEC) to the hippocampus in female AD mice.

**Conclusion:**

we reveal a female-specific APOE ε4-driven molecular network linking endothelial dysfunction to tau pathology. These hub genes provide potential biomarkers, while vincamine represents a targeted prevention and therapeutic candidate for high-risk APOE ε4-positive women.

## 1 Introduction

Alzheimer’s disease (AD), the leading cause of dementia, imposes a catastrophic socioeconomic burden with limited disease-modifying therapies (Scheltens et al., 2021). Although research has identified multiple genetic factors and biomarkers associated with AD pathogenesis, therapeutic development still faces persistent challenges regarding efficacy and safety (Graff-Radford et al., 2021; Khan et al., 2020). Decades of arduous efforts underscore the need for strategic pivots toward precision medicine and preventive interventions, which may yield greater clinical benefits while reducing long-term costs (Ferretti et al., 2018; Isaacson et al., 2018; Jucker and Walker, 2023).

The APOE gene exists in three primary variants (epsilon 2, epsilon 3, epsilon 4), among which the epsilon 4 (ε4) allele representing the strongest genetic risk factor for late-onset AD (Serrano-Pozo et al., 2021). APOE ε4 influences multiple pathological processes, including neuroinflammation (Nakata and Shinagawa, 1986), lipid metabolism (Raulin et al., 2022), amyloid-beta (Aβ) aggregation (Kaji et al., 2024), impairing Aβ clearance (Kaji et al., 2024), promoting amyloid deposition (Wisniewski and Drummond, 2020), and accelerating tau propagation (Chinnathambi et al., 2025). Consequently, elucidating how APOE modulates core AD pathologies is thus critical for developing therapies applicable to most patients.

Compelling epidemiological evidence reveals that women exhibit higher AD prevalence (e.g., post-65 prevalence is twice that of men) and accelerated cognitive decline, particularly post-menopause, indicating potent interaction between sex biology and genetic susceptibility (Ferretti et al., 2018). Analysis of data from the International Alzheimer’s Disease Coordinating Center by Altmann et al. showed that female ε4 carriers exhibited a greater risk of progressing to mild cognitive impairment or AD than their male counterparts, both among controls and individuals with mild cognitive impairment. This sex-specific effect remained statistically significant among controls and mild cognitive impairment patients when the analysis was restricted to APOE ε3/ε3 and APOE ε3/ε4 genotypes (Altmann et al., 2014; Scheyer et al., 2018). Moreover, for a given level of AD pathology, female APOE ε4 carriers may show greater levels of pathological burden, more compromised brain network integrity, and/or accelerated longitudinal decline (Altmann et al., 2014; Heise et al., 2014; Damoiseaux et al., 2012). Importantly, women also show heightened tau pathology independent of comparable Aβ burden (Coughlan et al., 2025). Although cardiovascular risks contribute, the biological basis for this sex dimorphism remains unresolved (Shufelt et al., 2018). Critically, despite well-established sex differences in AD manifestation, the mechanisms by which APOE genotypes differentially shape neural pathophysiology across sexes—particularly at the systems biology level remains elusive (Belloy et al., 2023).

Transcriptomic approaches provide an unbiased strategy to decode sex-specific APOE ε4-driven network perturbations, bridging genetic risk with actionable targets for prevention and treatment (Kelava et al., 2022). Here, we employ WGCNA to identify sex- and APOE ε4-linked disease modules. Through integrating Mendelian Randomization and machine learning approaches, we pinpointed hub genes and further validated their relationship with tau pathology. Single-cell RNA sequencing further reveals endothelial dysfunction as a key mechanism underlying sex-dimorphic APOE ε4 effects. Ultimately, we propose a mechanism-informed prevention strategy targeting high-risk APOE ε4-positive women, advancing precision medicine for vulnerable populations.

## 2 Materials and methods

### 2.1 Data collection and processing

*Human Cohorts*: RNA-seq BAM files, clinical features (including cognitive or neuro-pathological traits, sex and APOE genotype), mRNA expression data of Mayo cohorts and the Mount Sinai/JJ Peters VA Medical Center Brain Bank (MSBB) cohorts were obtained from the Accelerating Medicines Partnership Program for Alzheimer’s Disease^1^ and datasets of GSE15222 and GSE48350 were sourced from Gene Expression Omnibus (GEO) database.^2^ *Mouse Cohorts*: Female P301S tauopathy mice expressing human APOE ε3 or ε4 and matched controls (dataset GSE254103) were sourced from GEO database. All bulk-seq data were processed using R language (v4.3.1) and the fold change between different groups were calculated. Identification of differentially expressed genes (DEGs) was performed using the R package, “limma” (v3.56.2). Four cognitive or neuropathological traits are available in the MSBB dataset: CDR, Braak stage, neuropathology category as measured by crenezumab anti-amyloid in Alzheimer’s Disease (CERAD) (NP) and mean neocortical plaque density (plaque mean). Mayo subjects were separated into two groups (control and AD) based on neuropathological evaluation and classification. Control subjects had a Braak stage of ≤3, CERAD neurotic and cortical plaque densities of 0 (none) or 1 (sparse), and the absence of any of the following pathologic diagnoses: Parkinson’s disease, dementia with Lewy bodies, vascular dementia, postsynaptic potential, motor neuron disease, corticobasal degeneration, Pick’s disease, Huntington’s disease, frontotemporal lobar degeneration, hippocampal sclerosis, or dementia lacking distinctive histology. AD subjects have “definite AD” diagnosis according to the National Institute of Neurological and Communicative Disorders and the Stroke- Alzheimer’s Disease and Related Disorders Association (NINCDS-A DRDA) criteria and a Braak NFT stage of 4 or greater (Sekiya et al., 2023). For the correlation analysis, we leveraged the comprehensive clinical characterizations available in the MSBB dataset-including the CERAD score for amyloid-*β*, the Braak score for neurofibrillary tangle staging, and cognitive and functional assessments (CDR)-to stratify subjects into three distinct pathological progression groups. Specifically, samples were classified as: healthy control (HC, defined as Braak < 5, CDR < 3, and CERAD ≠ 1); mild-to-moderate cognitive impairment (MCI, defined as CERAD = 1, or Braak ≥ 5, or CDR ≥ 3); and late Alzheimer’s disease (LAD, defined as CERAD ≠ 4, Braak ≥ 5, and CDR > 0.5). This stratified approach enables a more direct comparison of hub gene changes across defined disease stages, offering clearer insights into their roles throughout the pathological continuum.

Single-cell RNA-seq data from 24 control and 24 Alzheimer’s disease samples (AMP-AD syn18485175) were processed using Seurat (v4.3.0.1) in R. The workflow initiated with quality control: genes expressed in ≥3 cells and cells containing ≥200 detected genes were retained, while cells exceeding 25% mitochondrial content (calculated via Percentage Feature Set) were excluded. Gene expression was normalized using Normalize Data, followed by identification of the top 2000 highly variable genes with FindVariableFeatures. Batch effects were corrected using Harmony integration and dimensionality reduction via Principal Component Analysis (PCA). Subsequent clustering utilized Harmony-corrected embeddings, with results visualized via t-SNE (Run tSNE). Finally, cell types were annotated based on canonical markers from the CellMarker database.^3^

### 2.2 WGCNA and hub genes

To enhance the quality of the co-expression network and reduce noise, initial gene filtering was performed by calculating the Median Absolute Deviation (MAD) for each gene across expression profiles, excluding the bottom 60% of genes with lowest MAD. Outlier genes and samples were subsequently removed using the goodSamplesGenes function in the R WGCNA package. A scale-free co-expression network was then constructed through the following workflow: First, pairwise Pearson’s correlation matrices were generated with average linkage hierarchical clustering. A weighted adjacency matrix was defined as:

A_mn=∣C_mn∣β

(C_mn = Pearson’s correlation between Gene_m and Gene_n, A_mn = adjacency between Gene m and Gene n). Then, select an appropriate soft-thresholding parameter to emphasize strong correlations while penalizing weak associations. This adjacency matrix was transformed into a Topological Overlap Matrix (TOM) quantifying network connectivity, with corresponding dissimilarity computed as D = 1 − TOM. Genes were clustered via average linkage hierarchical clustering based on TOM dissimilarity, applying a minimum module size of 30 genes and deepSplit sensitivity parameter of 3. Module eigengene dissimilarity was calculated to prune the dendrogram, with modules exhibiting dissimilarity < 0.25 being merged. The MSBB dataset initially contained 42,469 genes, which were reduced to 16,988 genes for WGCNA following median absolute deviation filtering. A soft-power threshold of 7 was selected according to the standard WGCNA scale-free topology criterion, aiming for a scale-free topology fit index (*R*^2^) exceeding 0.85. This approach, which has been widely adopted in the WGCNA literature (Langfelder and Horvath, 2008), balances network connectivity with a biologically meaningful structure. This process yielded 31 co-expression modules, wherein the “grey” module represented unassigned genes. For hub gene identification, Gene Significance (GS) was computed as the correlation between gene expression and clinical traits, while Module Membership (MM) measured the correlation between gene expression and module eigengenes. Based on the established thresholds (|MM| > 0.8 and |GS| > 0.1), 315 genes exhibiting high connectivity within the most relevant Female AD patient with_APOE:ε3/ε4 gene modules—*tan*, *blue2*, *grey60*, and *antiquewhite4*—were identified as hub genes.

### 2.3 Protein–protein interaction and biological function pathway enrichment analyses

PPI analyses were conducted by String.^4^ Gene Ontology (GO) pathway enrichment analysis was conducted by DAVID.^5^

### 2.4 Differential expression analysis

Limma (linear models for microarray data) is a method for screening differential expression genes based on the generalized linear model. Here, we use the R software package limma (v3.56.2) to conduct differential analysis to obtain the differentially expressed genes between different comparison groups and the control group. Specifically, for the expression profile dataset we obtained, we use the lmFit function to perform multiple linear regression and further use the eBays function to compute the moderated t-statistic, moderated F-statistic, and log-odds of differential expression by empirical Bayes moderation of the standard errors toward a common value. Finally, we obtain the differential significance of each gene. There are significant differences when *p < 0.05* and Fold change >1.2. For all analyses involving multiple comparisons, appropriate multiple testing corrections were applied using the Benjamini-Hochberg procedure to control the False Discovery Rate (FDR). The selection of an FDR threshold (0.05 and 0.1) represents a balance between statistical stringency and discovery power. A less stringent threshold (FDR = 0.1) was employed to enhance the detection of a broader set of putative findings while maintaining a controlled proportion of false discoveries (Ritchie et al., 2015). A heatmap of the hub genes and a volcano plot were generated using SANGERBOX.^6^

### 2.5 External validation of hub genes

The AlzData database^7^ was used to depict the expression of hub genes in multiple brain tissues of AD patients.

### 2.6 eQTL data for SMR analysis

In the summary-data-based Mendelian randomization (SMR) analysis, cis-expression quantitative trait loci (eQTLs) served as instrumental variables (IVs) for gene expression. Cis-eQTLs were defined as genetic variants located within ±5 Mb of the transcription start site (TSS) of target genes, with a default cis-window of 2 Mb applied for robustness. eQTL summary statistics were sourced from the eQTLGen consortium.^8^ To enhance statistical power for detecting brain-relevant eQTLs, we performed meta-analysis using the Meta-analysis of Cis-eQTLs in Correlated Samples (MeCS) method, which accounts for sample correlations through a random-effects model.

The SMR framework tested for causal associations by integrating gene expression and complex traits by leveraging cis-eQTL effects. We implemented the heterogeneity in dependent instruments (HEIDI) test to distinguish true pleiotropy from linkage disequilibrium (LD)-driven false positives. Heterogeneity In Dependent Instruments (HEIDI) is a method which seeks to address if many single nucleotide polymorphisms (SNPs) in a single region give Wald ratio (or ratio) estimates that are more different from each other than expected by chance under the assumption that each SNP only exhibits an effect due to linkage disequilibrium (LD) with the causal SNP. PHEIDI > 0.05 is considered to indicate no heterogeneity. Conversely, a significant HEIDI test (PHEIDI< 0.05) indicates that multiple independent causal variants may be present, rendering the SMR estimate unreliable (Sun et al., 2024; Xu et al., 2023). The Mendelian randomization (MR) method is shown in Supplementary Table S5.

### 2.7 Machine learning and core genes

Seven machine learning algorithms were adopted to further filter 315 hub genes for distinguishing between the female control group carrying the ε3/ε4 and female AD patients carrying the ε3/ε4: random forest (RF), least absolute shrinkage selection and Selection Operator (LASSO), Support Vector Machine (SVM), K-Nearest Neighbors (KNN), eXtreme Gradient Boosting (XGB), Gradient Boosting Machine (GBM), C5.0 and Neural Network (NNET) (Wu et al., 2024). RF is an ensemble prediction method that can handle many input variables and evaluate the importance of variables. We performed LASSO regression with the R packages (glmnet v4.1–8). SVM (R package: e1071 v1.7–14): Kernel-based classification with radial basis function. KNN (R package: class v7.3–22): Instance-based learning (k = 5 optimized via grid search). XGBoost (R package: xgboost v1.7.5): Gradient boosting with tree ensembles (learning rate = 0.01, max depth = 3). GBM (R package: gbm v2.1.8.1): Stochastic gradient boosting (shrinkage = 0.1, trees = 100). C5.0 (R package: C5.0 v0.1.8): Rule-based decision tree with boosting (trials = 10). NNET (R package: nnet v7.3–19): Single-hidden-layer neural network (size = 5, decay = 0.01). The diagnostic power of the final gene set was quantified using receiver operating characteristic (ROC) curves for all seven models. Area under the curve (AUC) was computed via 10-fold cross-validation (R package: pROC). The genes that consistently ranked in the top five across these multiple, independent classifiers were designated as the “core genes.”

### 2.8 The next-generation Cmap (CMap2)

The team behind the original Connectivity Map (CMap) subsequently developed its next-generation iteration (Subramanian et al., 2017), CMap2,^9^ as part of the NIH Library of Integrated Network-Based Cellular Signatures (LINCS) initiative. The LINCS-L1000 database substantially expands the original CMap, profiling responses to over 19,000 small molecules and nearly 5,000 genetic perturbagens (e.g., single-gene over-expression or knockdown) across an expanded panel of cell lines. We first identified gene modules significantly correlated (*p < 0.05*) with the FAD_APOE:ε3/ε4 trait, which yielded four positive modules (*antiquewhite4*, *tan*, *blue2*, *grey60*) and one negative module (*honeydew1*). We extracted genes meeting our hub gene criteria (|MM| > 0.8 and |GS| > 0.1), resulting in 315 positively correlated and 40 negatively correlated hub genes. From these pre-filtered hub genes, we selected the top 150 positively correlated genes and due to the smaller modules with significant negative correlations, all 40 negatively correlated genes, ranked by the absolute value of their Gene Significance (|GS|) for CMap analysis. This ensures the analysis was driven by the hub genes with the strongest statistical association to our key trait. This CMap database utilizes pattern-matching algorithms to compare input gene signatures with gene signatures in diverse contexts of over 450,000 chemical perturbagens (Chen et al., 2021).

### 2.9 Stereotaxic AAV injection

Six-month-old female APP/PS1 (C57BL/6 strain) mice were obtained from Jiangsu Huachuang Sino PharmaTech Co., Ltd. The mice were group-housed with littermates under a 12 h light/dark cycle with ad libitum access to food and water. All animal procedures were conducted in compliance with the Institutional Animal Care and Use Committee guidelines at China Pharmaceutical University (approval no. 2024-10-122). The pAAV-hSyn-MAPT(P301L)-HA-T2A-EGFP-WPRE (AAV- hTau, AAV9) vectors were purchased from OBiO Technology (Shanghai) Corp., Ltd. We injected 1 × 10^9^ AAV particles bilaterally into the MEC (layer II/III) using stereotaxic coordinates: anteroposterior −4.75 mm, lateral +2.9 mm, and dorsoventral −4.6 mm. Histological staining was performed 35 days post-injection.

### 2.10 Vincamine administration

A vincamine (MedChemExpress) dose of 30 mg/kg (*n* = 4) was administered intraperitoneal injected to treatment groups. The warm normal saline and 1 M citric acid (80:20, v/v, pH 3.5) was used as injection solvent (Juan and Tsai, 2005). Administration started on the day of AAV-hTau injection, once a day for 35 consecutive days. The control group was given a blank solvent.

### 2.11 RT-qPCR

Brain cortex tissues from APP/PS1_AAV-hTau and 30 mg/kg vincamine-treated APP/PS1_AAV-hTau mice were homogenized, and RNA was extracted with TRIzol reagent. Reverse transcription employed Vazyme’s R323 kit, while PCR amplification used the Q341 kit, performed on an Applied Biosystems GeneAmp PCR System 9,700 and a Roche Light Cycler 480 II, respectively. The RT-PCR primer sequences were: *Lrp10* (Forward: GGATCACTTTCCCACGTTCTG, Reverse: GAGTGCAGGATTAAATGCTCTGA); *Lamc1* (Forward: TGCCGGAGTTTGTTAATGCC, Reverse: CTGGTTGTTGTAGTCGGTCAG); *Rbms2* (Forward: ATGCTGCTATCAGTGACTTCCA, Reverse: GTCCTCGGATATACAGGTTGGT); *Ltbp2* (Forward: AAGCACCAACCACTGTATC, Reverse: CCTGGCATTCTGAGGGTCAAA); *Tmod3* (Forward: CTCCTTGGCAAGCTGTCCG, Reverse: CCCGTGGCAGACTTTGATGT) and *Gapdh* (Forward: TGGATTTGGACGCATTGGTC, Reverse: TTTGCACTGGTACGTGTTGAT). Melting curve analysis demonstrated PCR specificity through single-peak profiles. Relative RNA expression levels were quantified via the 2^(^−ΔΔCt^) method using Gapdh for normalization. Intergroup comparisons relied on *t*-tests, with significance thresholds set at *p < 0.05, **p < 0.01, and ***p < 0.001.

### 2.12 Immunohistochemical staining

The tissue sections and slides were deparaffinized and hydrated. The antigens were heat-retrieved, followed by tissue blocking; overnight primary antibody (Phospho-Tau (Ser202, Thr205;1:150); MN1020 ThermoFisher) (Zhu et al., 2022) incubation at 4 °C; secondary antibody incubation; and incubation with ABC reagent. The slides were scanned using the Fluorescence Inversion Microscope System (Olympus, stemi 2000 C) analyzed in ImageJ. Intergroup comparisons relied on *t*-tests, with significance thresholds set at *p < 0.05, **p < 0.01, and ***p < 0.001.

## 3 Results

### 3.1 Identification of APOE ε4- and sex-associated co-expression modules via weighted gene co-expression network analysis

**Figure 1:**
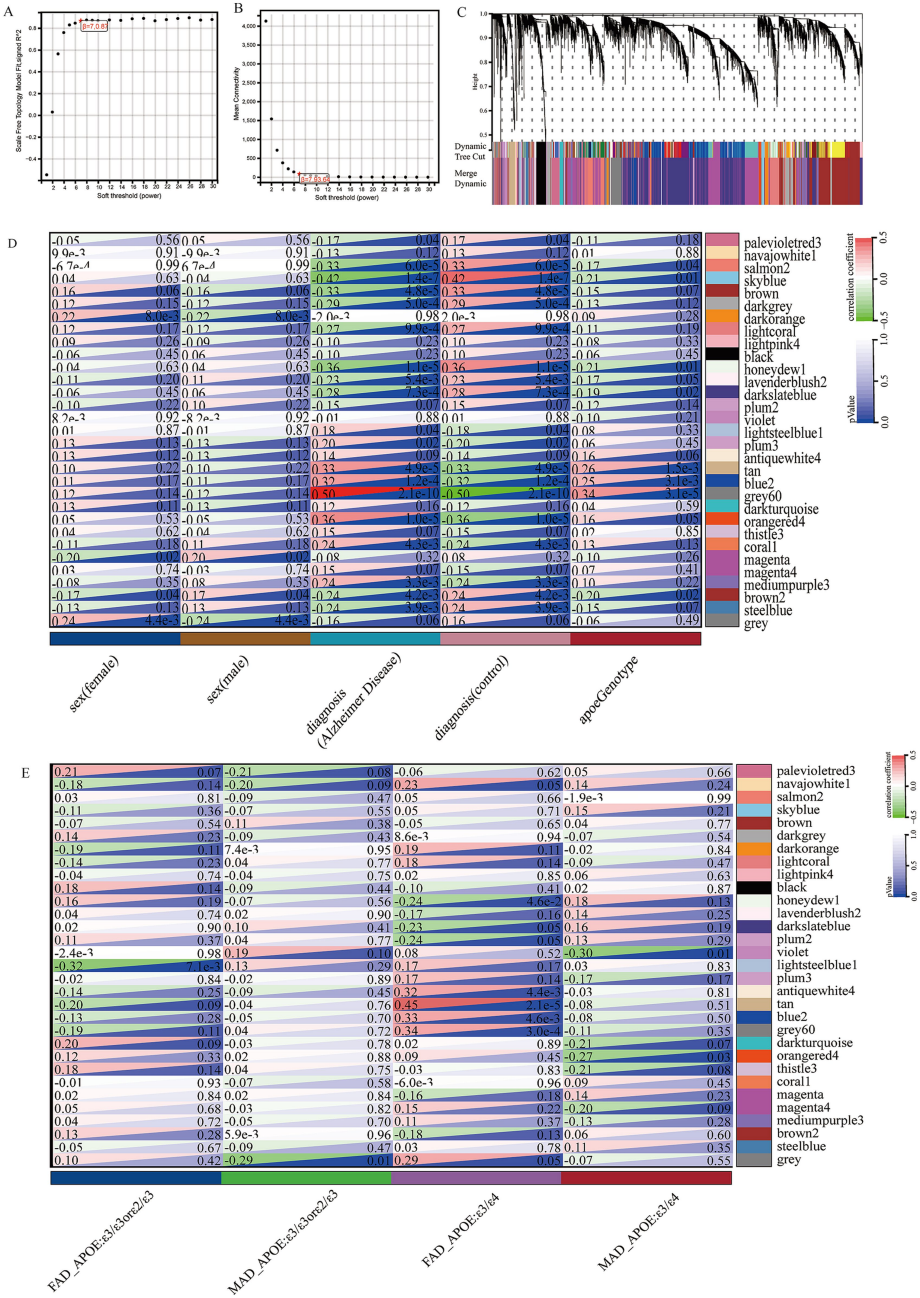
Construction of weighted gene co-expression networks. **(A)** Choosing the best soft-threshold power. **(B)** Analysis of the average connectivity of 1–30 soft threshold power. **(C)** Gene dendrogram and modules before and after merging. **(D)** Pearson correlation analysis of merged modules and sex, diagnosis and APOE genotype. **(E)** Pearson correlation analysis of merged modules and FAD_APOE (ε3/ε3 or ε2/ε3), MAD_APOE (ε3/ε3 or ε2/ε3), FAD_APOE (ε3/ε4), and MAD_APOE (ε3/ε4).

**Figure 2:**
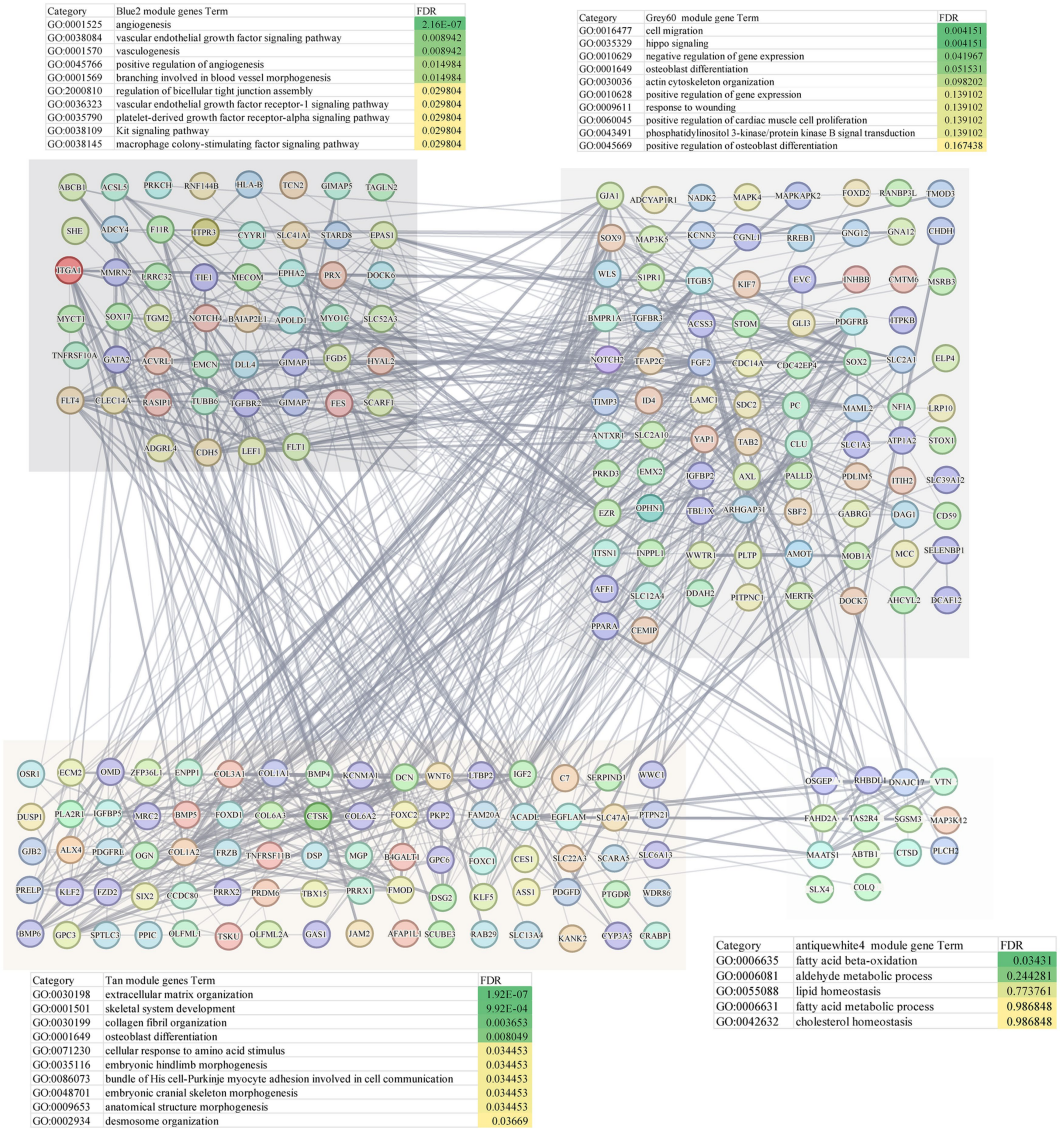
PPI network of hub genes and GOBP analysis.

WGCNA was applied to construct a scale-free co-expression network using RNA-seq data from 277 temporal cortex samples in the Mayo cohort. The soft-threshold power in this research was calibrated to 7 (scale-free R2 = 0.87; Figures 1A,B), resulting in the identification of 31 co-expression modules and dendrogram clearly shows that the four key female-specific modules (*antiquewhite4*, *tan*, *blue2* and *grey60*) reside on distinct branches (Figure 1C; Supplementary Table S1). Among these, four modules (*tan*, *blue2*, *grey60* and *orangered4*) showed significant positive correlations with AD pathology (correlation coefficient > 0.3, *p <* 0.01), while four modules (*salmon2*, *skyblue*, *brown* and *honeydew1*) exhibited significant negative correlations (Figure 1D). Notably, we found that the *tan*, *blue2* and *grey60* modules positively correlated with the APOE genotype associated with AD susceptibility (risk rank: ε4 > ε3 > ε2) (Lanfranco et al., 2020). To further explore the cross-relationship between gene modules—APOE ε4 and sex, we constructed correlation heatmap between four phenotypes and gene modules: FAD_APOE:ε3/ε3 or ε2/ε3 (female AD patient with APOE ε3/ε3 or ε2/ε3 genotype), MAD_APOE:ε3/ε3 or ε2/ε3 (male AD patient with APOE ε3/ε3 or ε2/ε3 genotype), FAD_APOE:ε3/ε4 (female AD patient with APOE ε3/ε4 genotype), and MAD _APOE:ε3/ε4 (male AD patient with APOE ε3/ε4 genotype) (Figure 1E). Strikingly, modules *antiquewhite4*, *tan*, *blue2* and *grey60* correlated exclusively with the female AD patient carrying APOE ε4, however there was no significant correlation with the phenotypes of the other sex/APOE genotype combinations. This finding aligns with the elevated AD risk conferred by APOE ε4 and underscores the need to explore these modules’ biological functions to explain female-specific susceptibility. Through Gene Ontology Biological Process (GOBP) analysis, the *blue2* gene module is mainly enriched in angiogenesis, vascular endothelial growth factor signaling pathway, angiogenesis and other signaling pathways, and the *grey60* gene module is mainly enriched in biological processes such as cell migration, hippo signaling and negative regulation of gene expression. The *tan* gene module is mainly enriched in biological processes such as extracellular matrix production, skeletal system development and glial fiber production, and the *antiquewhite4* gene module is mainly enriched in fatty acid *β*-oxidation, acetaldehyde metabolism processes and lipid homeostasis (Figure 2). Hub genes from clinically significant, female-specific APOE ε4-associated modules were identified using cutoffs of |MM| > 0.8 and |GS| > 0.1, yielding 315 high-connectivity hub genes (Figure 2; Supplementary Table S1). The protein–protein interaction (PPI) network for hub genes derived from the female-specific APOE ε4-associated modules is presented in Figure 2. Given the substantial overlap between the gene modules significantly associated with AD pathology (Figure 1D) and those specifically correlated with female APOE ε4 carriers (Figure 1E), subsequent analyses focused on the hub genes identified within the female-specific APOE ε4-associated modules (*antiquewhite4*, *tan*, *blue2*, *grey60*).

To validate the association between gene modules and sex or APOE genotype, we performed an independent WGCNA on the GSE15222 dataset. This analysis identified the yellow and brown modules as significantly linked to the FAD_APOE:ε3/ε4 phenotype (Supplementary Figure 1A). The genes in these modules were all enriched for the vascular endothelial signaling pathway (Supplementary Figures 1B,C).

### 3.2 Hub genes exhibit female-specific overexpression in APOE ε4 AD carriers and correlate with AD progression

**Figure 3:**
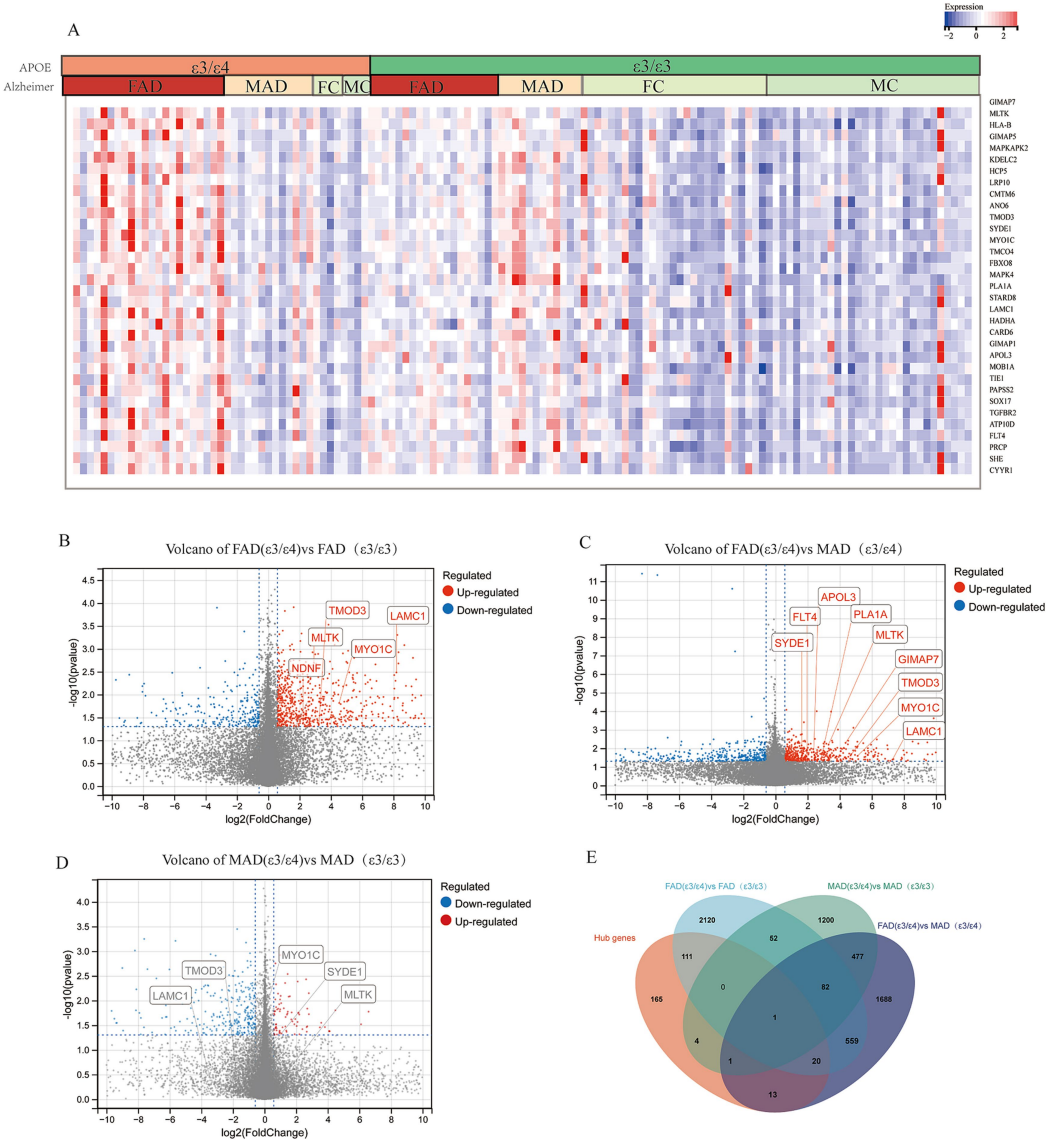
Hub genes from core modules were overexpressed in female AD patients with APOE ε3/ε4 in mayo cohort. **(A)** Heatmap of gene expression of representative hub genes, Female AD patients (FAD), Male AD patients (MAD), FC (Female Control), MC (Male Control). **(B)** Volcano plots illustrating the genes expression of FAD (APOE ε3/ε4) compared to FAD (APOE ε3/ε3). **(C)** Volcano plots illustrating the genes expression of FAD (APOE ε3/ε4) compared to MAD (APOE ε3/ε4). **(D)** Volcano plots illustrating the genes expression of MAD (APOE ε3/ε4) compared to MAD (APOE ε3/ε3). **(E)** Venn diagrams to show the numbers of DEGs identified in different groups of comparison: FAD (APOE ε3/ε4) versus MAD (APOE ε3/ε4); MAD (APOE ε3/ε4) versus MAD (APOE ε3/ε3); FAD (APOE ε3/ε4) versus FAD (APOE ε3/ε3).

Expression analysis revealed significant sex-specific patterns in mayo cohort. In female patients, many hub genes exhibited significantly elevated expression in APOE ε3/ε4 AD cases compared to healthy controls with APOE ε3/ε4, and the 33 representative genes shown in the heatmap drawn from the 315 hub genes that showed significant upregulation in the female AD (ε3/ε4) subgroup (*p < 0.05*, Figure 3A); specifically, 132 of 315 hub genes were overexpressed in female APOE ε3/ε4 AD patients versus female APOE ε3/ε3 AD patients (Figures 3B,E). Conversely, only 6 of 315 hub genes showed significant differential expression in male patients between APOE ε3/ε4 and ε3/ε3 AD groups (*p < 0.05*; Figures 3D,E). Furthermore, female APOE ε3/ε4 AD patients demonstrated significantly higher expression levels of hub genes compared to male APOE ε3/ε4 AD patients (Figures 3C,E). Consistent findings were also evident following Benjamini-Hochberg false discovery rate correction (Supplementary Figure 3). Relative to female and male APOE ε3/ε3 AD patients, female APOE ε3/ε4 AD patients exhibited elevated expression levels of several hub genes, including *MLTK*, *LAMC1*, *LRP10*, *MAPKAPK2*, *GIMAP5*, *KDELC2*, *HCP5* and *HLA-B* (FDR < 0.1), with *MLTK*, *LAMC1* and *GIMAP5* reaching a more stringent significance threshold (FDR < 0.05). Analysis of the GSE15222 dataset further revealed that 23 of the 315 hub genes, including *STARD8*, *GIMAP5*, *APOL3*, *PTGDR*, *LRP10*, *TMOD3* and *HLA-B*, exhibited significantly higher expression in the FAD (ε3/ε4) group compared to the FAD (ε3/ε3) and MAD (ε3/ε4) groups, which are among the 33 representative genes shown in Supplementary Figures 2A,B. Similar expression trends for these representative genes were also observed in the GSE48350 and MSBB datasets (Supplementary Figures 2C,D). To rule out confounding effects from altered cellularity, we performed two complementary analyses: cell proportion analysis revealed no significant differences in endothelial cell abundance between key groups, and pseudobulk differential expression analysis confirmed the significant upregulation of endothelial genes (including *LAMC1, LRP10,* and *TMOD3*) specifically in FAD_APOE:ε3/ε4 samples, indicating a genuine cell-intrinsic transcriptional response rather than proportional shifts (method in Supplementary Table S5; Supplementary Figures 4A,B).

**Figure 4:**
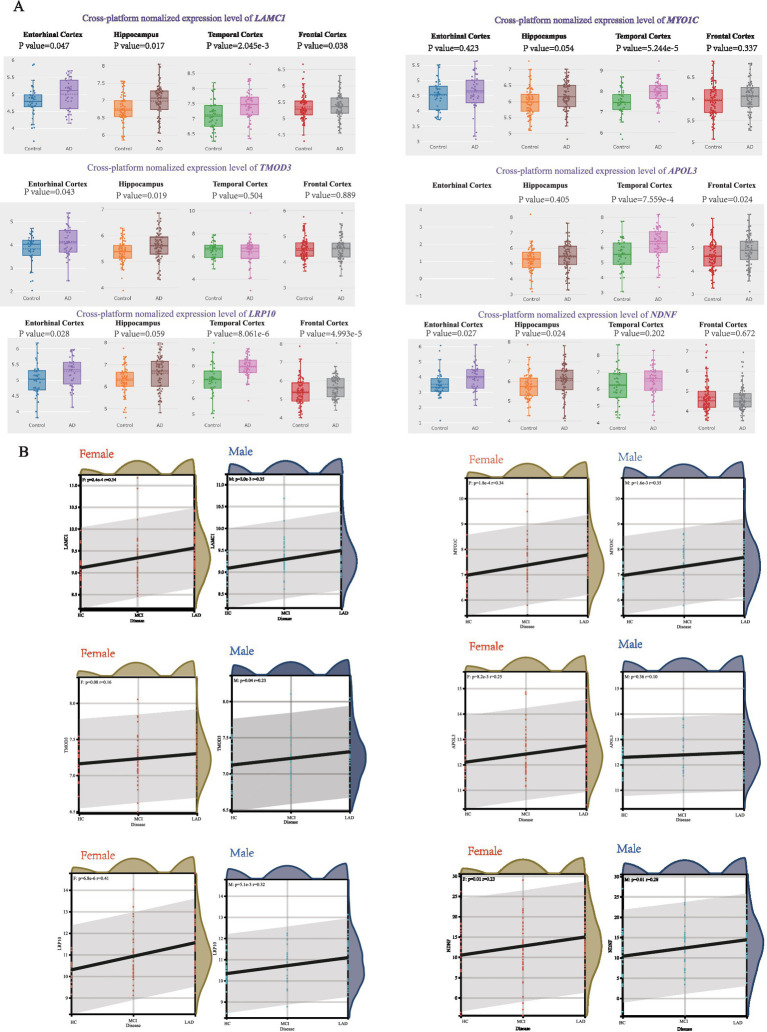
Correlation between hub genes and pathological status of AD. **(A)** AlzData database showing the expression levels of *LAMC1*, *MYO1C*, *TMOD3*, *APOL3*, *LRP10* and *NDNF* in AD patient and control group. **(B)** The correlation between female/male AD pathology and *LAMC1*, *MYO1C*, *TMOD3*, *APOL3*, *LRP10*, *NDNF* expression in MSBB cohort.

To validate the association of these hub genes with AD progression, we analyzed expression patterns across those brain regions using the AlzData database. Six hub genes-*LAMC1*, *MYO1C*, *TMOD3*, *APOL3*, *LRP10*, and *NDNF*—were consistently upregulated in AD patients across multiple brain regions (Figure 4A). We further assessed their correlation with pathological severity in the MSBB cohort (BM22 RNA-seq datasets), stratifying samples into three groups: Healthy Controls (HC), mild to moderate pathological lesions (MCI), and Late-stage AD (LAD) based on CERAD, Braak, and CDR scores. *LAMC1*, *MYO1C*, *LRP10*, and *NDNF* expression showed a significant positive correlation with AD progression in both men and women, *TMOD3* showed a significant positive correlation with AD progression in men, and *APOL3* showed a significant positive correlation with AD progression in females (Figure 4B). Other hub genes exhibited similar correlations (Supplementary Figure 5).

### 3.3 Integrating SMR for causal gene identification and machine learning for diagnostic gene mining

To identify causal genes underlying AD pathogenesis from modules highly correlated with FAD (ε3/ε4) group (*salmon2*, *skyblue*, *brown* and *honeydew1*), we performed Summary-data-based Mendelian randomization (SMR) integrating GWAS and eQTL data, applying thresholds of PSMR<0.01 and PHEIDI>0.05 on 315 hub genes, we identified 10 genes with causal associations to AD progression (Table 1), including *RBMS2*, *RFX2*, *KLF5*, *TMOD3* and *LTBP2*. Most importantly, to strengthen robustness and address tissue-specificity concerns, we performed independent Mendelian Randomization analyses using two independent datasets—FinnGen R9 (G6_ALZHEIMER) and ieu-b-2. This validation successfully confirmed the potential causal role of key genes (e.g., *RBMS2* and *TMOD3*), thereby reinforcing the reliability of our findings across multiple data sources (Supplementary Figure 6).

**Table 1:** SMR results from eQTL.

Probe	Gene	Top SNP	PeQTL	PGWAS	Beta	SE	PSMR	PHEDI
ENSG00000076067	RBMS2	rs3782235	0	0.02	0.064152	0.027592	0.0201	0.438
ENSG00000087903	RFX2	rs74792734	8.51E-48	0.0185	−0.32702	0.140613	0.02	0.531
ENSG00000102554	KLF5	rs9573281	8.78E-78	0.0182	−0.23195	0.099013	0.0191	0.759
ENSG00000105538	RASIP1	rs1231281	4.43E-24	0.023	−0.44767	0.201856	0.0266	0.298
ENSG00000115825	PRKD3	rs10177970	0	0.0345	−0.11174	0.052925	0.0347	0.012
ENSG00000116016	EPAS1	rs11691694	5.3E-182	0.00532	−0.19586	0.07061	0.00554	0.598
ENSG00000119681	LTBP2	rs754458	7.06E-62	0.0419	0.225521	0.111661	0.0434	0.695
ENSG00000132357	CARD6	rs74445588	1.02E-40	0.00374	0.354257	0.125042	0.00461	0.243
ENSG00000133065	SLC41A1	rs823075	7E-157	0.000538	0.256749	0.074807	0.000599	0.0204
ENSG00000138594	TMOD3	rs16964596	2.77E-71	0.0445	−0.21419	0.107255	0.0458	0.662

**Figure 5:**
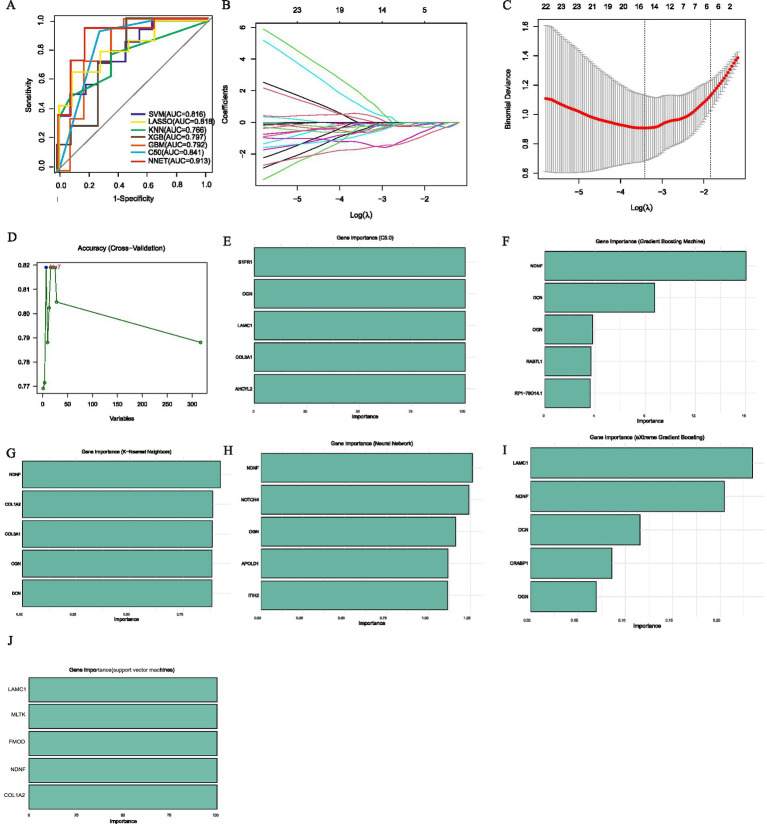
Machine learning to identify diagnostic genes of female AD patients with APOE ε3/ε4. **(A)** ROC curves from seven models. **(B)** LASSO coefficient profiles of candidate genes. **(C)**. Cross-validation to select the optimal tuning parameter log (Lambda) in LASSO regression analysis. **(D)** SVM Recursive Elimination Validation. **(E)** Top five important trait genes of C5.0. **(F)** Top five important trait genes of GBM. **(G)** Top five important trait genes of KNN. **(H)** Top five important trait genes of NNET. **(I)** Top five important trait genes of XGB. **(J)** Top five important trait genes of SVM.

Concurrently, we employed seven machine learning methods, we initially screened out 315 hub genes, and then employed (SVM, LASSO, KNN, XGB, GBM, C5.0, NNET) to mine diagnostic genes differentiating APOE ε3/ε4 female healthy controls from APOE ε3/ε4 female Alzheimer’s disease patients. After an initial screening of 315 relevant genes, dimensionality reduction models were applied to identify key diagnostic genes. The area under the receiver operating characteristic curve (AUC) values of the ROC curves of SVM, LASSO, KNN, XGB, GBM, C5.0 and NNET were 0.816, 0.818, 0.766, 0.797, 0.792, 0.841 and 0.913, respectively (Figure 5A). The core genes identified by the LASSO model include *DOCK7*, *ACOX1*, *COL3A1*, *MLTK*, and *ACSL5* (Figures 5B,C), and the C5.0 model includes *S1PR1*, *OGN*, *LAMC1*, *COL3A1*, and *AHCYL2* (Figure 5E). The core genes of the GBM model include *NDNF*, *DCN*, *OGN*, and *RP1-78O14.1* (Figure 5F). The core genes of the KNN model include *NDNF*, *COL3A1*, *OGN*, and *DCN* (Figure 5G). The core genes of the NNET model include *NDNF*, *NOTCH4*, *OGN*, *APOLD1*, and *ITIH2* (Figure 5H); the XGB model’s core genes include *LAMC1*, *NDNF*, *DCN*, *CRABP1*, and *OGN* (Figure 5I); while the SVM model’s core genes include *LAMC1*, *MLTK*, *FMOD*, *NDNF*, and *COL1A2* (Figures 5D,J). In summary, genes such as *TMOD3* and *RBMS2* have a causal relationship with the occurrence and development of AD. Genes like *NDNF*, *LAMC1*, and *MLTK* are not only core module genes in WGCNA and the sex-specific transcriptomic signatures of the APOE ε4 allele but also core genes in various machine learning models predicting transition from FC to FAD in a female with APOE ε3/ε4.

### 3.4 Hub genes are associated with the pathological development of tau and are involved in the progression of cognitive dysfunction

**Figure 6:**
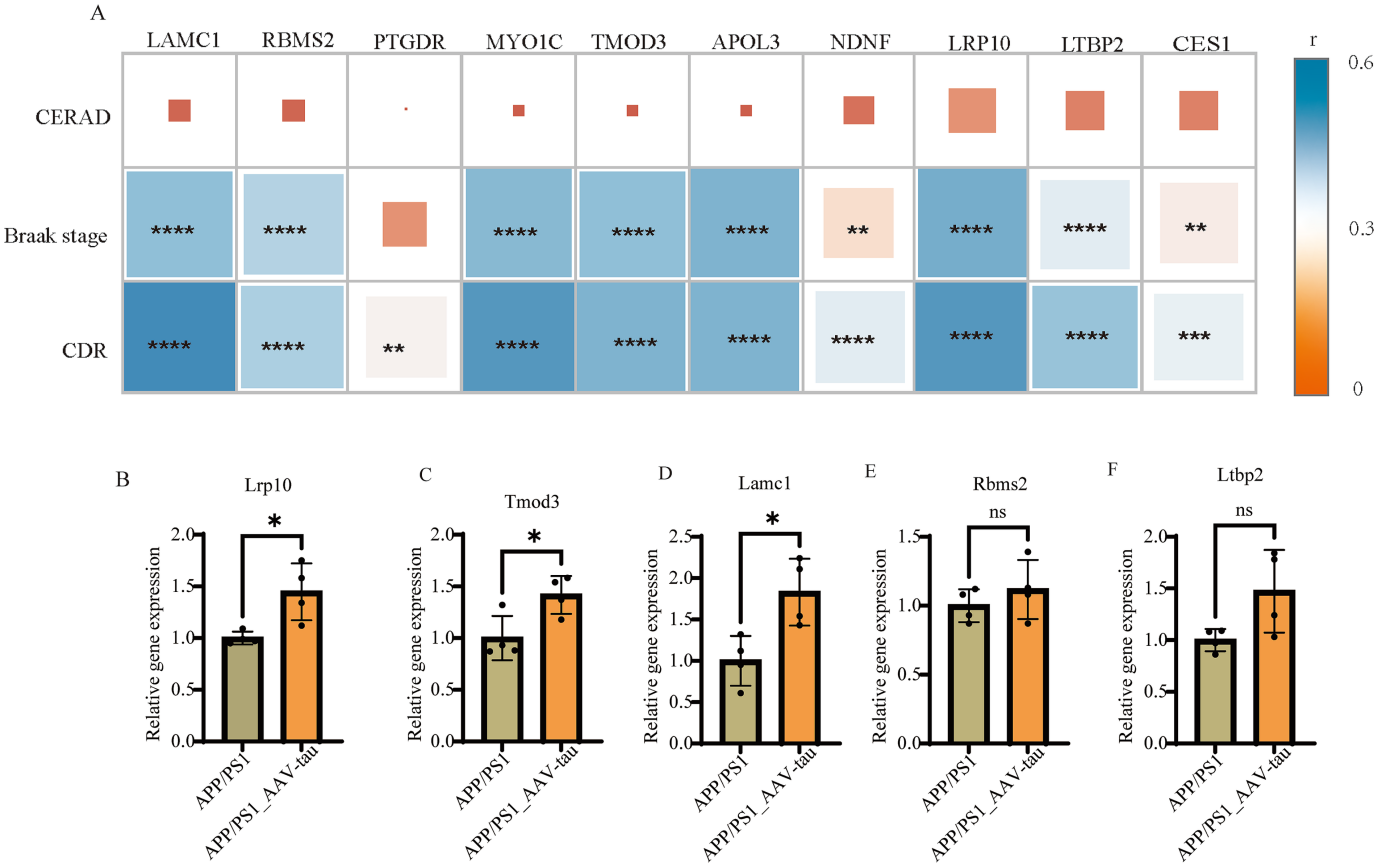
**(A)** Correlation analysis of gene expression with CERAD scores, Braak stages, and CDR scores in MSBB cohort. **(B–F)** Relative mRNA levels of *Lrp10*, *Tomd3*, Lamc1, *Rbms2* and *Ltbp2* of brain cortex, *n* = 4. *For *p* < 0.05. “ns” for no significant difference.

The primary neuropathological hallmarks of AD encompass amyloid plaques, neurofibrillary tangles, synaptic loss, and glial hyperplasia (Scheltens et al., 2021; Ossenkoppele et al., 2022). To investigate the relationship between hub gene and established AD pathological progression, we analyzed gene expression data from the MSBB cohort (BM36 region) alongside key neuropathological and clinical indices: Braak stage (reflecting NFT progression), CERAD score (assessing neuritic plaque density), and Clinical Dementia Rating (CDR) score. Braak Stages I and II display limited neurofibrillary tangles (NFTs) within the transentorhinal and entorhinal cortex and is often associated with normal cognitive and clinical profiles (Harding et al., 2000; Vanderlinden et al., 2025). Braak stage III and IV display NFTs in the hippocampus and neocortex adjoining the trans-entorhinal cortex refers to the limbic phase when limited cognitive dysfunction is initially observed (Therriault et al., 2022; Fleisher et al., 2020), labeled the neocortical phase, presents with the greatest number and extent of NFTs and is often associated with significant cognitive and functional impairment. CERAD score is a semiquantitative measure of neuritic plaques (Kivipelto et al., 2013). A neuropathologic diagnosis was made of no AD, possible AD, probable AD, or definite AD based on semi-quantitative estimates of neuritic plaque density as recommended by the Consortium to Establish a Registry for Alzheimer’s Disease (CERAD), modified to be implemented without adjustment for age and clinical diagnosis. A CERAD neuropathologic diagnosis of AD required moderate (probable AD) or frequent neuritic plaques (definite AD) in one or more neocortical regions. Neuropathological analysis revealed a similar prevalence of Braak stage III in individuals with a clinical dementia rating (CDR) (Muir et al., 2024). Clinical dementia rating scale (CDR) is conducted for assessment of dementia and cognitive status. Final diagnoses and CDR scores are conferred by consensus. Based on CDR classification, subjects are grouped as no cognitive deficits (CDR = 0), questionable dementia (CDR = 0.5), mild dementia (CDR = 1.0), moderate dementia (CDR = 2.0), and severe terminal dementia (CDR = 3.0–5.0). Our analyses revealed significant positive correlations between hub genes (*LAMC1*, *RBMS2*, *MYO1C*, *TMOD3*, *APOL3*, *NDNF*, *LRP10*, *LTBP2*, *CES1*) and established measures of Alzheimer’s disease progression, specifically Braak stage and CDR score. However, no significant association was observed with the CERAD score. This pattern suggests that the identified hub genes are likely to influence cognitive decline predominantly through mediating tau pathology propagation rather than via other pathways broadly assessed by the CERAD composite (Figure 6A).

To further validate the correlation results, we compared the mRNA expression levels of *Lrp10*, *Tmod3*, *Lamc1*, *Rbms2*, and *Ltbp2* in cortical tissues of female APP/PS1 mice and female AAV-hTau-injected APP/PS1 mice. The results demonstrated that intracerebral injection of hTau significantly increased the mRNA expression levels of *Lrp10*, *Tmod3*, and *Lamc1* (Figures 6B–D). Additionally, an upward trend in the expression of *Rbms2* and *Ltbp2* was also observed (Figures 6E,F).

### 3.5 Elevated expression of hub genes *LAMC1*, *RBMS2*, and *TMOD3* in endothelial cells of AD patient by single-cell analyses

**Figure 7:**
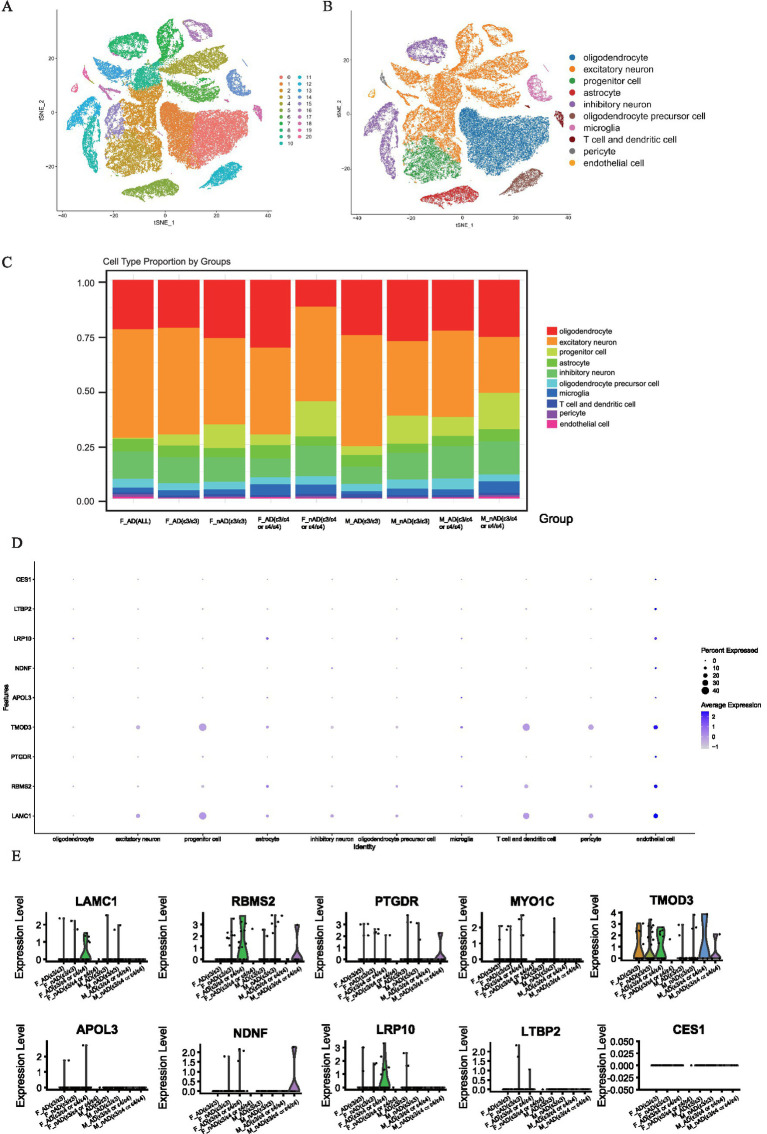
Single-cell analysis of gene expression in the brain tissues dataset syn18485175. **(A,B)** tSNE dimensionality reduction plot displaying the diverse cellular landscape in the dataset. **(C)** Proportion of all cell types in different groups. **(D)** Feature plot map of representative hub genes. **(E)** Violin plots showed the expression levels of *LAMC1*, *RBMS2*, *PTGDR*, *MYO1C*, *TMOD3*, *APOL3*, *NDNF*, *LRP10*, *LTBP2* and *CES1* in endothelial cell.

To investigate the potential role of hub genes within specific cell types, we analyzed the single-nucleus RNA-seq dataset (AMP-AD syn18485175) (Mathys et al., 2019). The t-SNE visualization robustly clustered major cell populations in the temporal cortex (Figure 7A), with clusters annotated using the CellMarker database (Figure 7B). We found that there were significant differences in the proportions of various cell types among samples with different disease states, genders, and apolipoprotein E (APOE) genotypes. Notably, the proportion of progenitor cell significantly decreases in all AD samples compared to no AD pathology samples, and we discovered that the proportion of oligodendrocyte in F_nAD (female with no AD pathology) (APOE ε3/ε4) was significantly lower than other groups (Figure 7C). Dot plots revealed broad expression of *TMOD3*, *RBMS2* and *LAMC1* expressed across progenitor cell, T cell and dendritic cell and endothelial cell (Figure 7D). Through violin plots, we found that in endothelial cells, the mRNA expression of *LAMC1*, *RBMS2*, *MYO1C*, *TMOD3*, *APOL3*, and *LRP10* in the F_AD (female with AD pathology) (APOE ε3/ε4 or ε4/ε4) group was much higher than other groups. Combined with the information on expression levels, the overall trend was consistent with the results of Bulk RNA-seq (Figure 7E). However, no similar trend was observed in neural precursor cells, T cells and presenting cells (Supplementary Figure 7).

**Figure 8:**
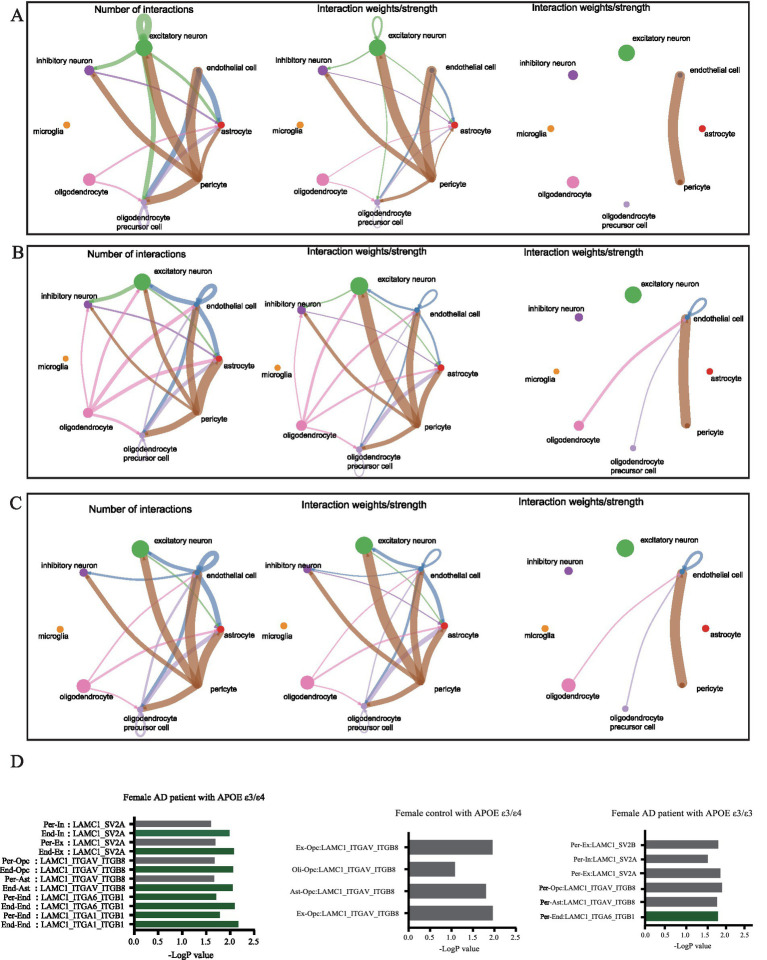
Cellular communication networks through single-cell transcriptomic analysis of dataset syn18485175. **(A)** Network diagram showing the number of interactions and the interaction strength between various cell types in control group with APOE ε3/ε4. **(B)** Network diagram showing the number of interactions and the interaction strength between various cell types in AD group with APOE ε3/ε3. **(C)** Network diagram showing the number of interactions and the interaction strength between various cell types in AD group with APOE ε3/ε4. **(D)** Bar chart showed the relative contribution of different ligand-receptor pairs in the ECM signaling pathway.

Analysis of intercellular signaling revealed distinct network patterns across cohorts, Non-AD APOE ε3/ε4 or ε4/ε4 females and APOE ε3/ε3 AD females exhibited balanced neuron-astrocyte-oligodendrocyte precursor communication networks; Conversely, APOE ε3/ε4 or ε4/ε4 female AD patients demonstrated three hallmark alterations: diminished neuron-to-glial signaling (Figures 8A,B), significantly enhanced endothelial communication, and elevated oligodendrocyte activity (Figures 8B,C). Extracellular matrix pathway interrogation specifically implicated endothelial Laminin B2 Chain (LAMC1)-ligand dysregulation in APOE ε4-carrying female AD patients (Figure 8D), where aberrant interactions with parenchymal ligands may disrupt endothelial homeostasis through integrin-mediated Mechan transduction pathways. These results establish a direct association between endothelial-specific gene dysregulation (particularly *LAMC1*), compromised neurovascular unit integrity, and tau pathology progression, highlighting actionable targets for precision interventions in this high-risk demographic.

### 3.6 Animal data were used to verify the expression of hub genes, as well as drug prediction and validation

**Figure 9:**
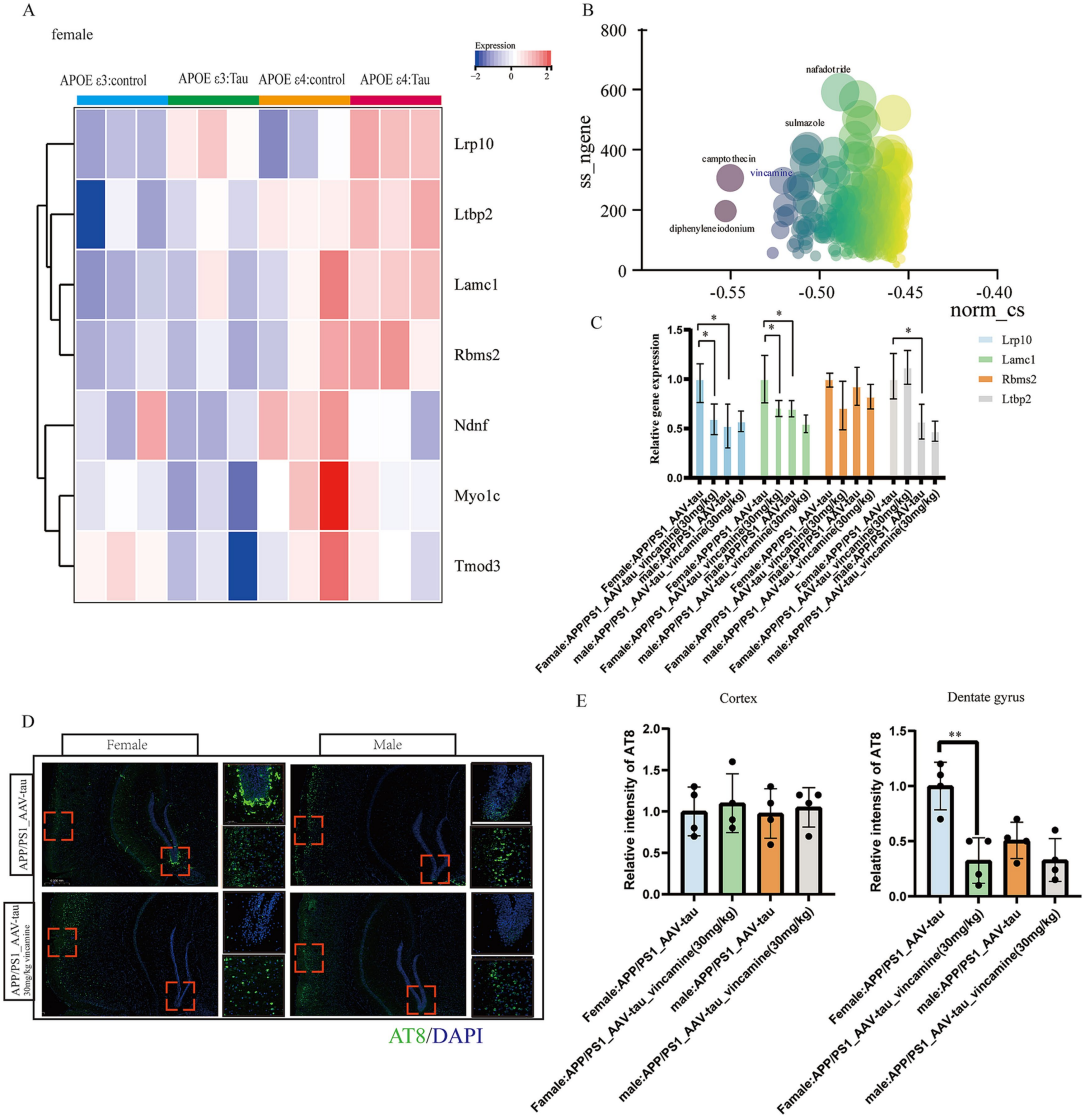
Animal data validation as well as drug prediction. **(A)** Heatmap of hub genes in GSE254103. **(B)** Connectivity map scoring bubble chart, **(C)** Relative mRNA levels of *Lrp10*, *Lamc1*, *Rbms2* and *Ltbp2* of brain cortex, *n* = 4. **(D,E)** Representative histological images and statistical chart in cortex and DG brain regions stained for AT8 (green), and nuclei (DAPI, blue) in APP/PS1 mouse brain 5 weeks following AAV-hTau injection, *n* = 3. Bar = 50 μm. * For *p < 0.05*, * * for *p < 0.01*.

To validate the regulatory role of APOE ε4 on hub genes and its impact on tau pathology, we extracted female P301S tauopathy mice RNA-seq data GSE254103 for further analysis. Heatmap analysis revealed that the human APOE ε4 allele significantly upregulated hub genes *Lrp10*, *Ltbp2*, *Lamc1*, *Rbms2* etc., and the expression levels of tauopathy mice with APOE ε4 were higher than those of control mice with APOE ε4, revealing that these genes were also key genes associated with tau pathology (Figure 9A). Subsequently, we utilized the 150 positively correlated genes from modules *antiquewhite4*, *tan*, *blue2* and g*rey60* and 40 negatively correlated genes from module *honeydew1* with the highest GS score to conduct drug repositioning analysis using the Connectivity Map (CMap). We identified vincamine, a vasodilator, as a top candidate that significantly counteracts the transcriptional profile of female APOE ε3/ε4 Alzheimer’s disease (AD) patients (Figure 9B). Besides, according to previous research, vincamine has a good ability to cross the blood–brain barrier (Juan and Tsai, 2005), so we considered vincamine may have the potential to ameliorate tau pathology by improving vascular endothelial cell function. Our results demonstrate that intraperitoneal administration of vincamine (30 mg/kg/day) significantly reduced cortical expression levels of *Lrp10* and *Lamc1* in female AAV-hTau-injected APP/PS1 mice (*p < 0.05*), and in female mice, vincamine treatment led to statistically significant downregulation of these target genes. In male mice, while a downward trend was often present, the changes frequently failed to reach statistical significance (Figure 9C). Moreover, vincamine treatment markedly attenuated the propagation of phosphorylated tau (P-Tau, AT8 immunoreactivity) within hippocampal tissues (*p < 0.01*). Notably, age-matched male mice exhibited significantly less hippocampal P-Tau propagation compared to females. Vincamine administration showed no significant effects on cortical or hippocampal P-Tau levels in male mice (*p > 0.05*, Figures 9D,E). Our study further confirms that vincamine ameliorates tau pathology in female murine models.

## 4 Discussion

This study integrates multi-omics approaches to delineate the molecular interplay between APOE ε4, sex-specific vulnerability, and AD pathogenesis. Our key findings reveal sex- and APOE ε4-Dependent transcriptional networks: WGCNA identified co-expression modules (e.g., *tan*, *blue2*, *grey60*) strongly correlated with AD pathology and particularly female patient with APOE ε4 genotype.

We identified three gene modules enriched in female APOE ε4 carriers with Alzheimer’s disease, each linked to distinct biological processes: the “blue2” module with angiogenesis, the “tan” module with extracellular matrix remodeling, and the “grey60” module with actin cytoskeleton organization. This enrichment indicates that these pathways may contribute to the increased AD susceptibility in this population. Next, we identified hub genes within gene modules that may drive female-specific AD pathogenesis. These hub genes—including *LAMC1*, *TMOD3*, *NDNF*, *RBMS2*, and *MYO1C*—showed increased expression only in female APOE ε4 carriers compared to female controls or ε3/ε3 AD patients. This expression pattern was not observed in males. Besides, these genes correlated with tau pathology progression (Braak stage, CDR) but not amyloid load (CERAD), implicating them in tau-mediated cognitive decline.

Both SMR and MR analyses revealed that genes such as *RBMS2* and *TMOD2* may be key causal genes for AD. Furthermore, in multiple machine learning models, hub genes including *LAMC1*, *NDNF*, *OGN*, and *COL3A1* demonstrated a significant association with disease risk. These findings suggest that these hub genes may hold considerable potential for AD diagnosis and treatment.

Single-cell validation confirmed vascular-endothelial dysfunction, with *LAMC1*, *RBMS2* and *TMOD3* upregulated in endothelial cells from female APOE ε4 Alzheimer’s disease brains. Altered intercellular crosstalk featured enhanced endothelial signaling and reduced neuronal communication, linking vascular dysfunction to tau pathology. APOE ε4 amplifies tau pathology via hub genes: In P301S tauopathy mice, female APOE ε4 boosted expression of *Lrp10*, *Ltbp2*, *Lamc1*, and *Rbms2*, revealing a potential link to the acceleration of tau-driven neurodegeneration. Drug repurposing nominated vincamine (vasodilator) as a potential therapeutic to rescue vascular-endothelial deficits in female APOE ε4 carriers. And our study further confirms that vincamine ameliorates tau pathology in female murine models.

Functionally, *LAMC1* encodes the γ1-chain of laminin, a key component of the extracellular matrix (ECM) that regulates blood–brain barrier (BBB) integrity (Wu et al., 2024) and neuronal adhesion. Emerging evidence indicates that laminin dysregulation compromises BBB tight junctions, increasing vascular permeability and facilitating the extravasation of neurotoxic plasma proteins (e.g., fibrinogen) into the brain parenchyma. This process may directly exacerbate neuroinflammation and accelerate tau pathology.

Our study identifies a robust, female-specific association between the APOE ε4 genotype and transcriptional alterations in brain endothelial cells. Building upon this correlation, we propose a plausible mechanistic model through which endothelial dysfunction may facilitate tau pathogenesis in female APOE ε4 carriers. We hypothesize that the APOE ε4 genotype triggers a cascade of events, including astrocyte dysregulation and microglial activation, which converge to disrupt endothelial homeostasis. This is substantiated by our findings of the significant upregulation of four hub genes—*LAMC1* (basement membrane integrity), *LRP10* (lipoprotein receptor signaling), *RBMS2* (RNA processing), and *TMOD3* (actin cytoskeleton regulation) (Ghosh et al., 2022)—all critical to vascular function. The concerted actions of these genes likely contribute to a compromised blood–brain barrier (BBB), which in turn may promote tau pathology via two interrelated pathways: (1) by creating a permissive environment for tau spread through impaired clearance of tau and potential facilitation of extracellular tau oligomer movement via a leaky barrier and extracellular vesicles (Fowler et al., 2025); and (2) by engaging in neuroinflammatory cross-talk, wherein BBB dysfunction perpetuates a pro-inflammatory cycle involving microglia and astrocytes (Liebner et al., 2018), which can directly induce tau hyperphosphorylation and stimulate its phagocytosis and release. While this model requires further causal validation, it provides a coherent, literature-grounded framework that links APOE ε4, sex-specific endothelial impairment, and tau propagation, moving beyond correlation to suggest a testable functional pathway. Our study focused on APOE ε4-driven effects, but other sex hormones (e.g., estrogen, FSH) (Xiong et al., 2022; Davis et al., 2021) or X-chromosome-linked factors may contribute to the observed transcriptional differences. While the hub genes identified in this study represent promising candidate biomarkers and mechanistic players derived from post-mortem brain tissue, their practical utility for early detection in living individuals requires future validation in accessible biofluids such as plasma or Cerebrospinal Fluid. Our work has begun to clarify the relationships between sex, APOEε4, Alzheimer’s disease, and vascular endothelial cells function. Further exploration of vascular alterations alongside genetic changes may broaden the utility of these hub genes for disease prediction and intervention. Integrative analyses with proteomic or epigenomic data could provide deeper mechanistic insights.

Vasoconstriction is a complex process primarily mediated by the contraction of vascular smooth muscle cells (VSMCs) (Touyz et al., 2018), while endothelial cells regulate vascular tone through the release of various factors (Miyauchi et al., 1990). When endothelial cells are damaged or dysfunctional, their ability to modulate vascular tone is compromised, leading to reduced bioavailability of vasodilatory factors such as nitric oxide (NO), along with increased production or enhanced action of vasoconstrictors such as endothelin-1 (ET-1), thereby promoting vasoconstriction (Kruger-Genge et al., 2019; Pepin and Gupta, 2024). Therefore, the vasoconstrictive effect associated with vincamine is relevant to our investigation of endothelial cell function. Furthermore, our previous gene enrichment analysis revealed that the hub genes used to target vincamine were enriched in signaling pathways related to angiogenesis (Su et al., 2011), extracellular matrix remodeling (Lemarie et al., 2010), and actin cytoskeleton reorganization (Yamin and Morgan, 2012)—processes associated with vasodilation. Thus, investigating the vasodilatory effects of vincamine represents an important direction in our study of how vincamine modulates the progression of Alzheimer’s disease.

Based on our findings and extant literature, we propose an integrated model wherein the female-specific APOE ε4 genotype drives endothelial dysfunction and blood–brain barrier (BBB) disruption (Jiang et al., 2025)—partly via upregulation of hub genes *LAMC1*, *LRP10*, *RBMS2*, and *TMOD3*—which in turn promotes tau pathogenesis through impaired tau clearance (Langfelder and Horvath, 2008; Xin et al., 2018; Yang et al., 2024) and neuroinflammatory crosstalk (Candelario-Jalil et al., 2022; Ising et al., 2019), collectively facilitating tau accumulation and spread.

## 5 Conclusion

We deciphered a female-specific gene network through which APOE ε4 accelerates AD via vascular dysfunction and tau pathology. Our hub genes offer novel biomarkers for early detection in high-risk females, while vincamine emerges as a mechanism-informed candidate therapy. This study contributes to the growing body of evidence supporting precision treatment and prevention of AD.

## Data Availability

The original contributions presented in the study are included in the article/Supplementary material, further inquiries can be directed to the corresponding authors.

## Footnotes

1
https://nia.nih.gov/research/amp-ad

2
https://www.ncbi.nlm.nih.gov/geo/

3
http://xteam.xbio.top/CellMarker/

4
https://cn.string-db.org/

5
http://david.abcc.ncifcrf.gov/

6
http://sangerbox.com/Index

7
http://www.alzdata.org/

8
https://eqtlgen.org/

9
https://clue.io
