# Supplementary material for: APOE ε4 allele drives female-specific Alzheimer’s disease progression via vascular dysfunction and tau spreading
Source: Front Neurosci. 2025 Dec 2;19:1683204. doi: 10.3389/fnins.2025.1683204 (PMC12705627; doi:10.3389/fnins.2025.1683204)
Supplement: Supplementary file 1 [file Data_Sheet_1.DOCX]

Supplementary Material

***Supplementary Material***

***
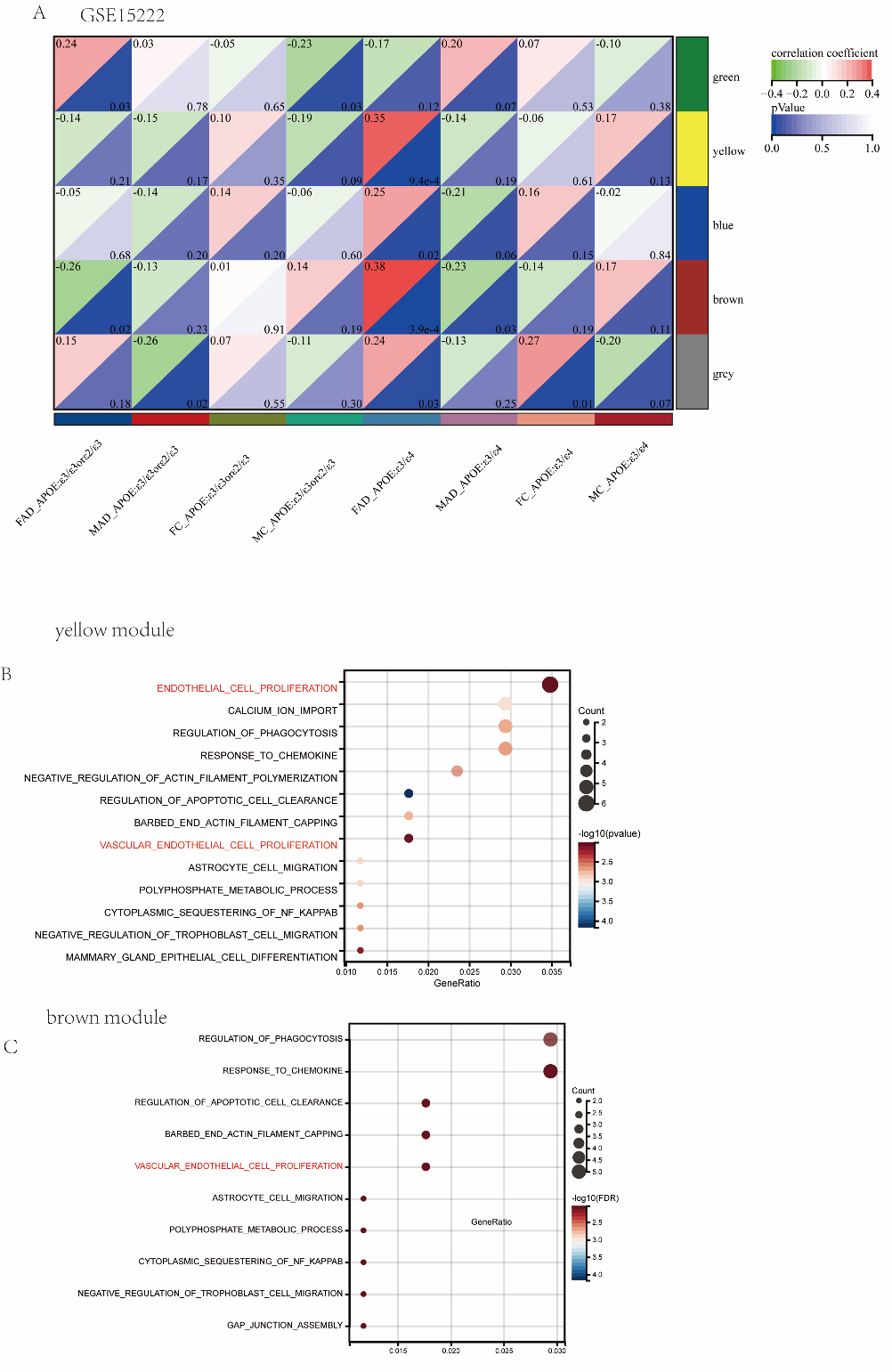
***

**Supplementary Figure 1.** Construction of weighted gene co-expression networks with GSE15222 datasets (A) Pearson correlation analysis of merged modules and FAD_APOE (ε3/ε3 or ε2/ε3), MAD_APOE (ε3/ε3 or ε2/ε3), FAD_APOE (ε3/ε4), MAD _APOE (ε3/ε4)，FC_APOE (ε3/ε3 or ε2/ε3), MC_APOE (ε3/ε3 or ε2/ε3), FC_APOE (ε3/ε4) and MC _APOE (ε3/ε4) ; (B) GOBP analysis of yellow gene module; (C) GOBP analysis of brown gene module.

***
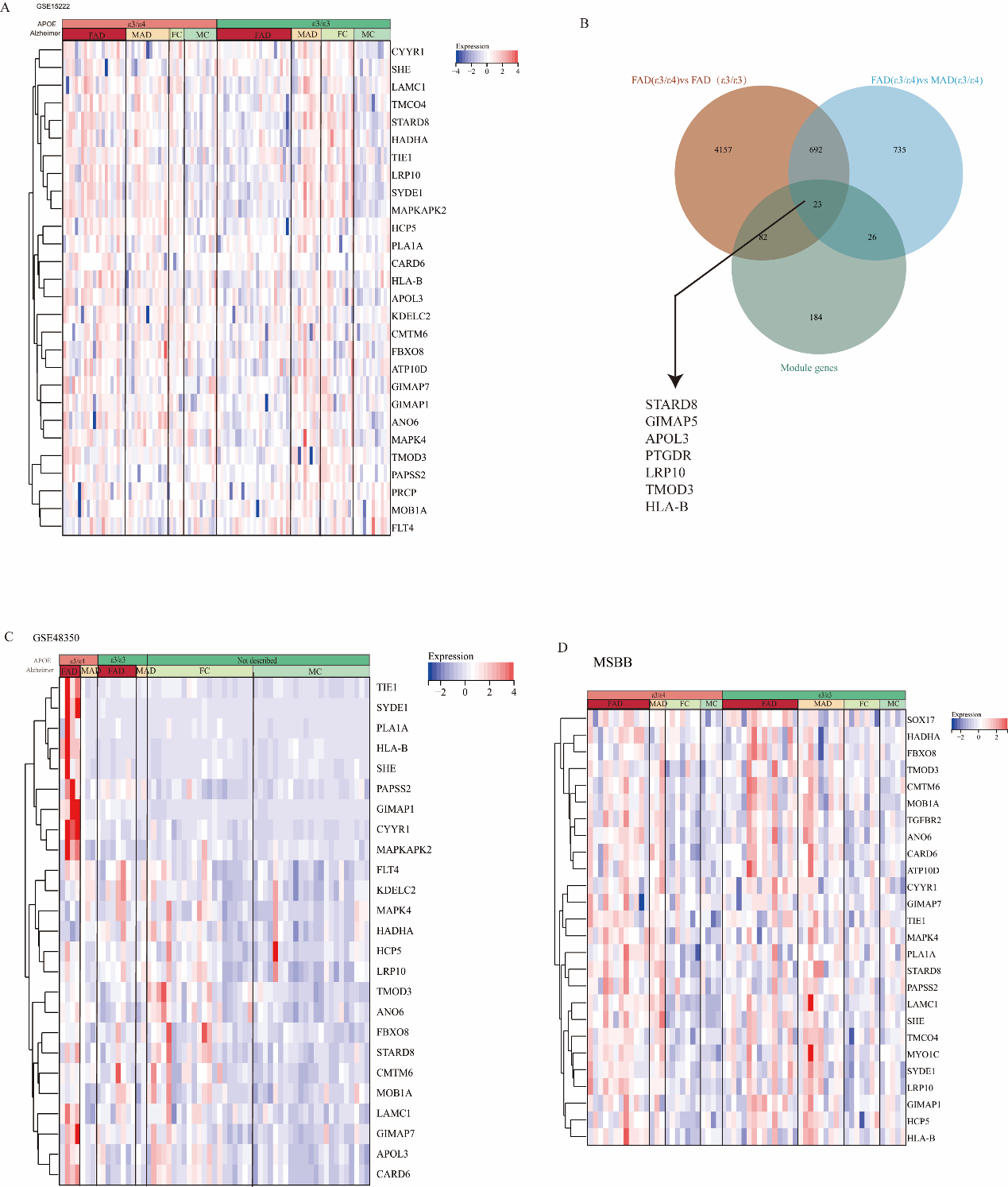
***

**Supplementary Figure 2.** Hub genes from core modules were overexpressed in female AD patients with APOE ε3/ε4. (A) Heatmap of gene expression of representative hub genes with GSE15222 datasets; (B) Venn diagrams to show the numbers of DEGs identified in different groups of comparison; (C) Heatmap of gene expression of representative hub genes with GSE48350 datasets; (D) Heatmap of gene expression of representative hub genes with MSBB (BM36) datasets


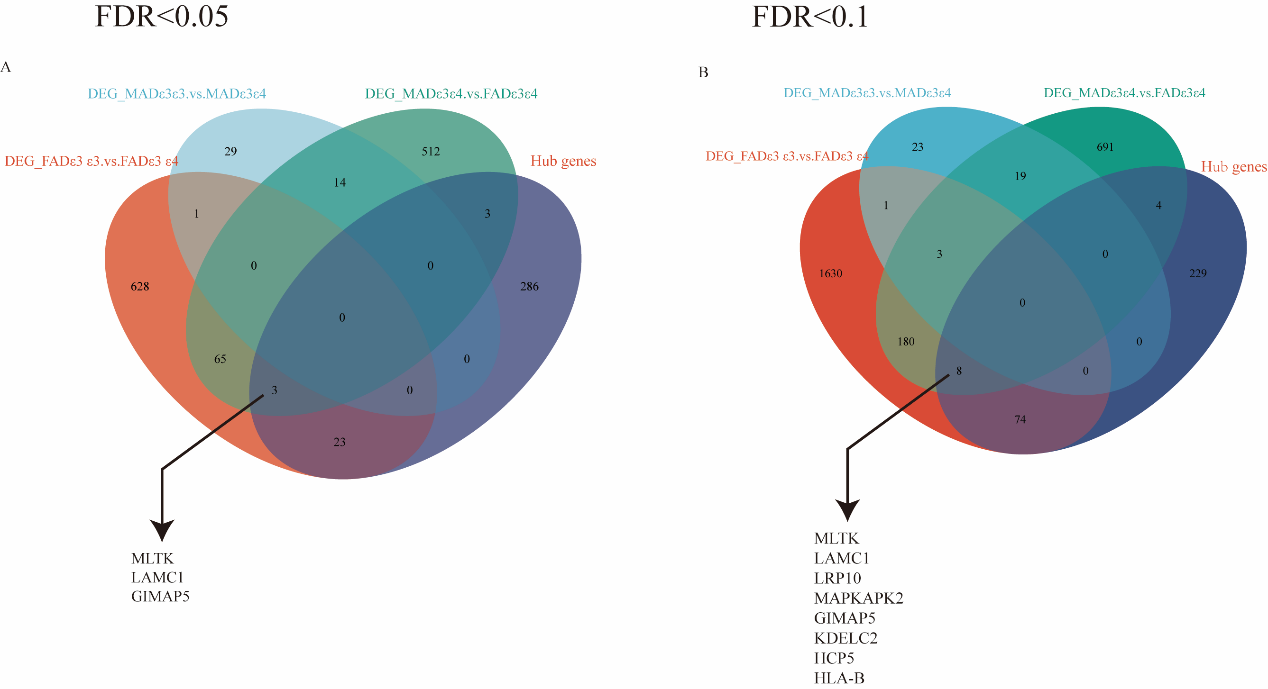


**Supplementary Figure 3.** Differential expression analysis of Mayo cohort datasets with False Discovery Rate. (A) Venn diagrams show the numbers of DEGs identified in different groups of comparison (FDR<0.05); (B) Venn diagrams to show the numbers of DEGs identified in different groups of comparison (FDR<0.1)


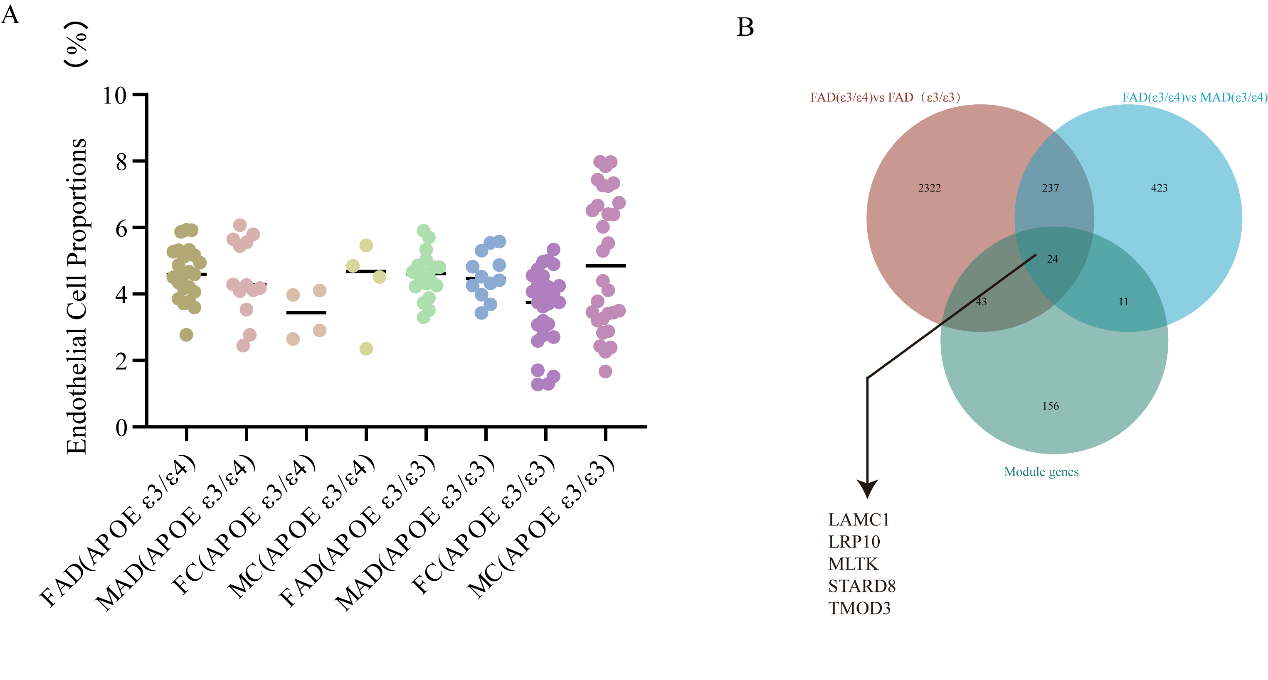


**Supplementary Figure 4.** Cell Composition Analysis and Pseudobulk Differential Expression Analysis in mayo cohort. (A) Endothelial cell proportions in different groups; (B) Venn diagrams to show the numbers of Pseudobulk DEGs identified in different groups of comparison (FDR<0.1)

***
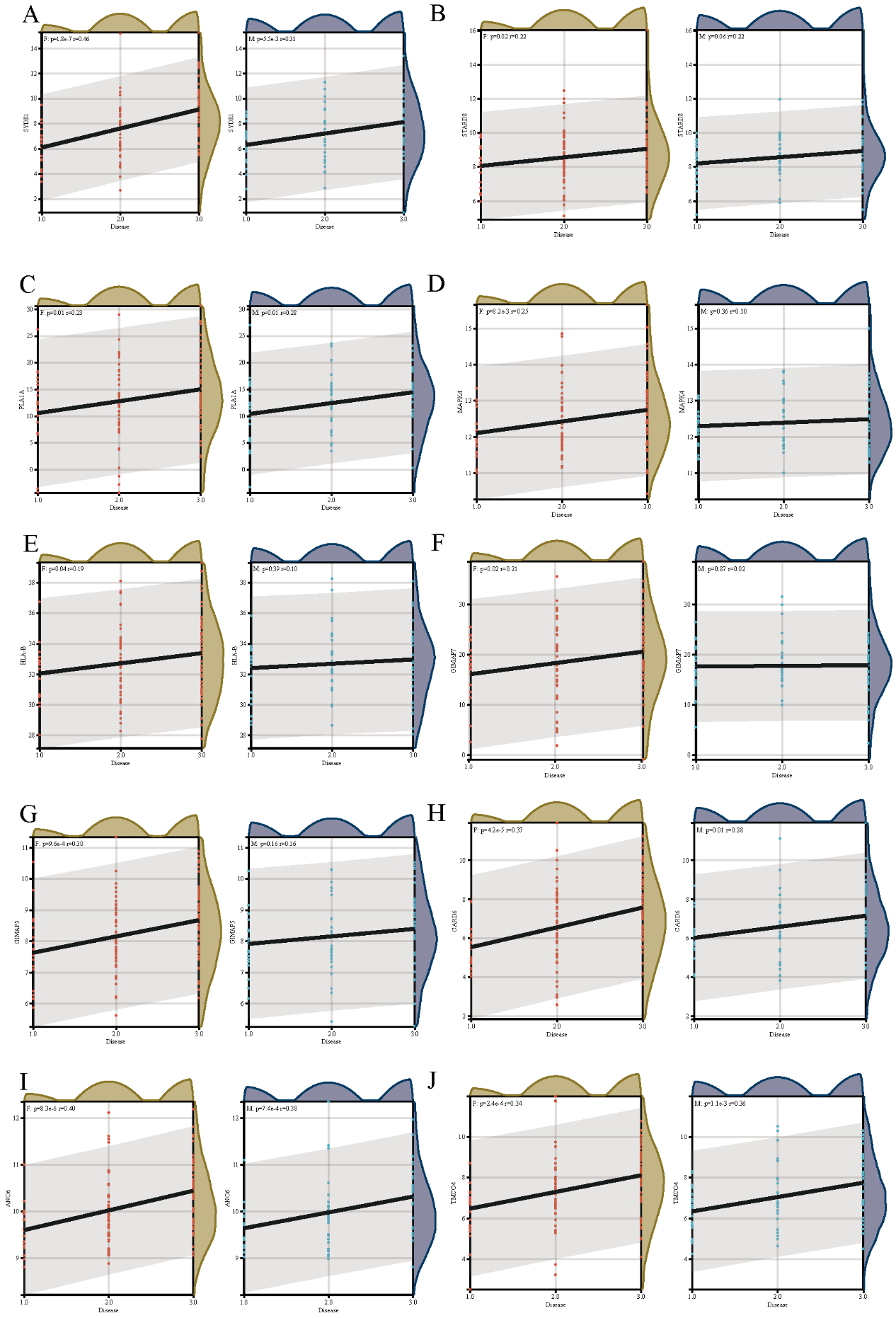
***

**Supplementary Figure 5.** The correlation between female/male AD pathology and SYDE1(A), STARD8(B), PLA1A(C), MAPK4(D), HLA-B(E), GMAP7(F), GMAP5(G), CAPD6(H), ANO6(I) andTMCO4(J) expression in MSBB cohort. The X-axis: 1 represents HC, 2 represents MCI, and 3 represents LAD.


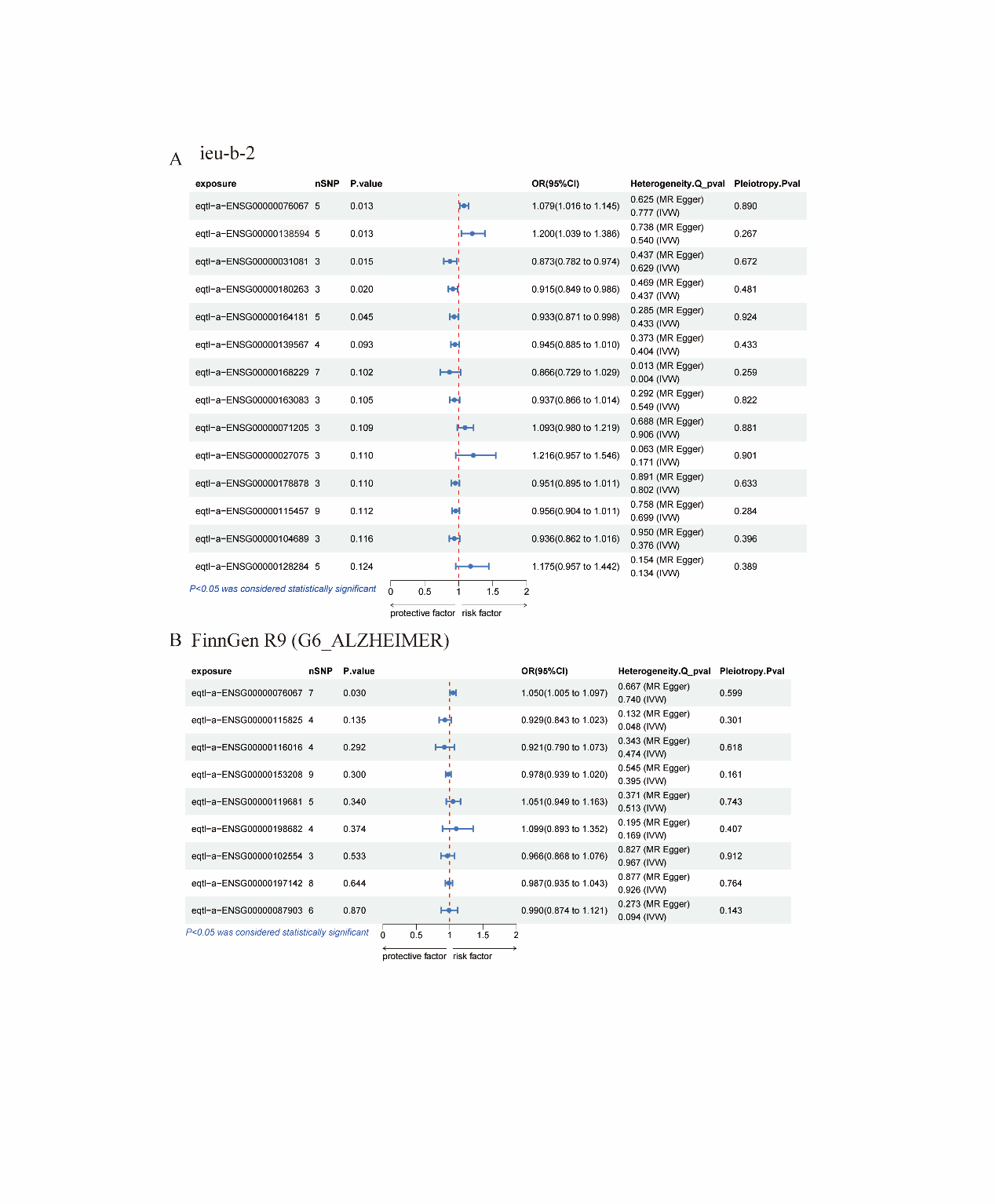


**Supplementary Figure 6.** Forest plot of the studies that evaluated the causal effect between hub genes and Alzheimer’s disease by the IVW MR method.(A) causal effect of hub genes on ieu-b-2; causal effect of hub genes on FinnGen R9(G6)


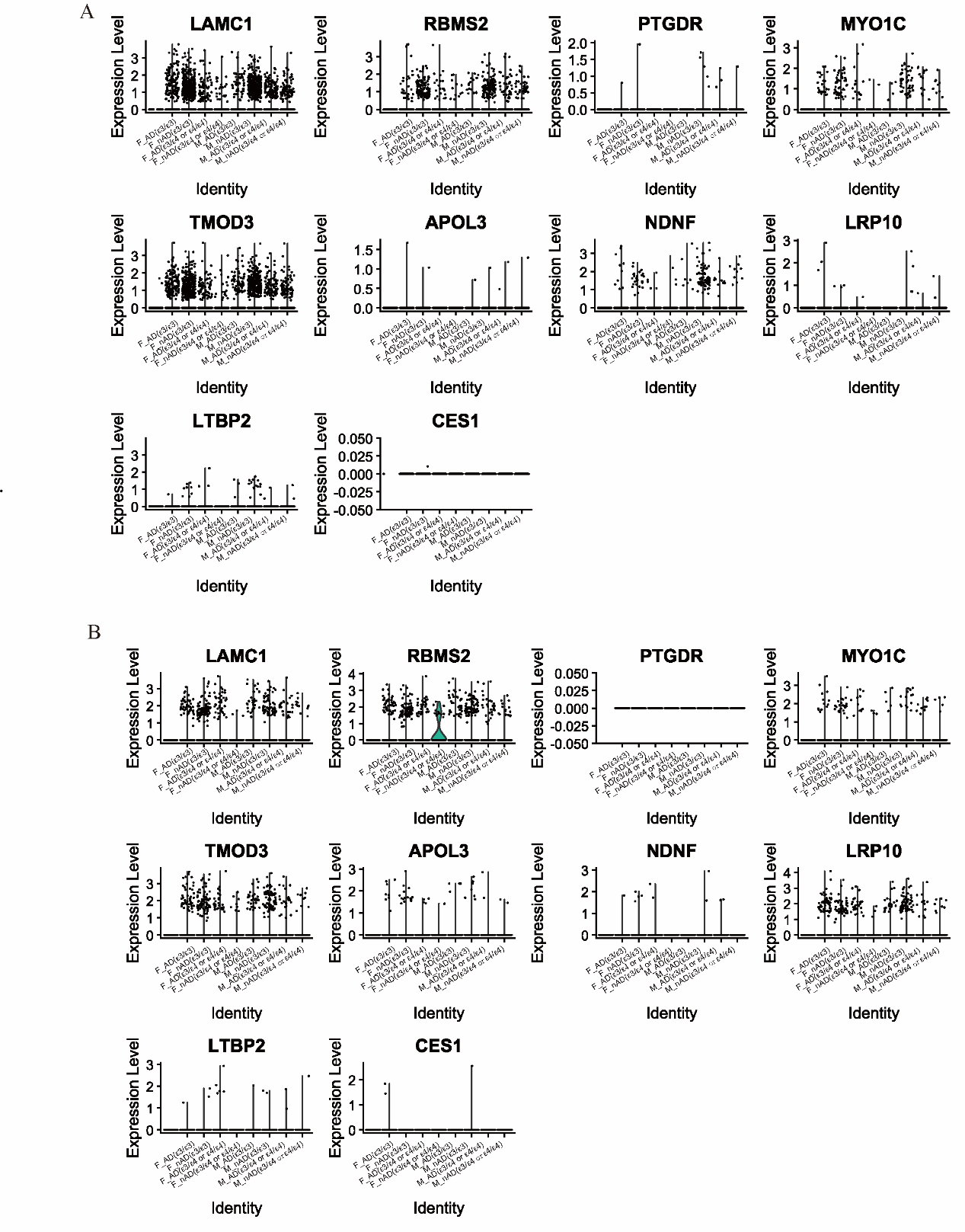


**Supplementary Figure 7** Violin plots showed the expression levels of LAMC1, RBMS2, PTGDR, MYO1C, TMOD3, APOL3, NDNF, LRP10, LTBP2 and CES1 in (A) progenitor cell and (B)T cell and dendritic cell


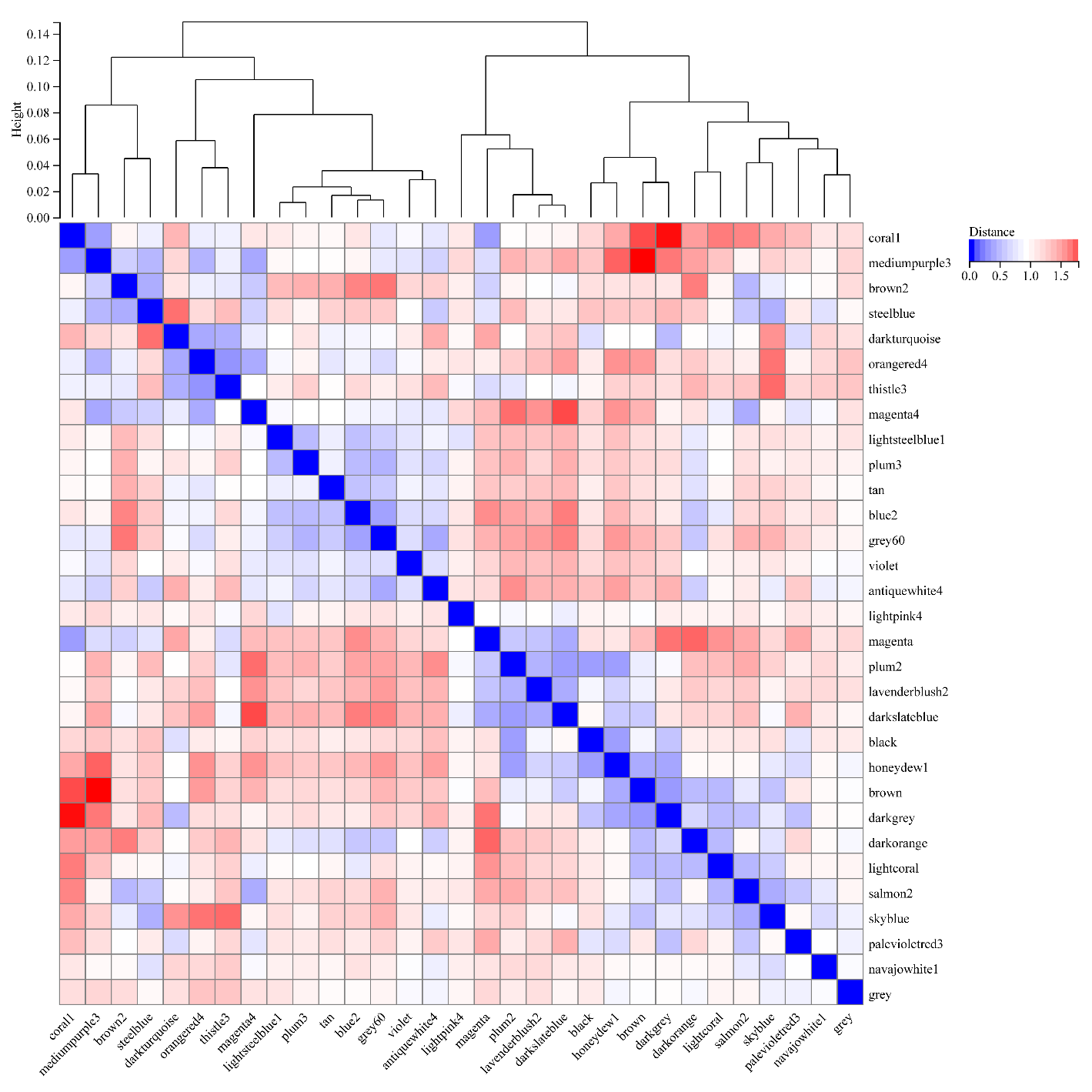


**Supplementary Figure 8** eigengene adjacency heatmap (The weighted gene co-expression network for the GSE15222 dataset)
